# Excited-State Intramolecular Proton Transfer: A TD-DFT Study of the Delayed Fluorescence Mechanism in Triquinolonobenzene

**DOI:** 10.1021/acs.jpca.6c04402

**Published:** 2026-10-27

**Authors:** Yuhui Liu, Mai-Ning Li, Si-Ying Lu, Yajing Peng, Yu Zhao, Jun Gao, Chaoyuan Zhu

**Affiliations:** † College of Physical Science and Technology, 12687 Bohai University, Jinzhou 121013, China; ‡ Hubei Key Laboratory of Agricultural Bioinformatics, College of Informatics, 47895 Huazhong Agricultural University, Wuhan 430070, P. R. China; § Department of Applied Chemistry, National Yang Ming Chiao-Tung University, Hsinchu 30010, Taiwan

## Abstract

The interplay between excited-state intramolecular proton transfer (ESIPT) and thermally activated delayed fluorescence (TADF) was investigated in the prototypical emitter triquinolonobenzene (TQB), using angular-quinacridone (*a*-QD), which exhibits analogous ESIPT behavior but lacks TADF, as a reference system. Excited-state transition-state optimizations reveal that ESIPT is essentially barrierless in both molecules. Owing to the negligible spin–orbit coupling (SOC), intersystem crossing (ISC) is ineffective prior to or during the proton-transfer process. Following ESIPT to the keto tautomer (TQB-TB), however, efficient ISC channels become available, with the calculated S_1_ → T_1_ and S_1_ → T_2_ rate constants reaching 1.10 × 10^8^ and 1.37 × 10^8^ s^–1^, respectively. More importantly, the exceptionally small S_1_–T_2_ energy gap (0.01 eV) enables highly efficient reverse ISC, resulting in a T_2_ → S_1_ rate constant of 9.70 × 10^7^ s^–1^, whereas the direct T_1_ → S_1_ rISC remains very slow (1.18 × 10^2^ s^–1^) owing to the much larger S_1_–T_1_ gap. Analysis of the vibronic coupling mechanism shows that Herzberg–Teller contributions dominate most ISC and reverse ISC processes, whereas the Franck–Condon contribution remains indispensable for the S_1_ ↔ T_2_ channels, accounting for 41.82% (S_1_ → T_2_) and 26.10% (T_2_ → S_1_) of the corresponding rates. These results establish a comprehensive mechanistic picture in which ESIPT promotes efficient TADF by activating favorable singlet–triplet interconversion pathways in the keto tautomer, providing molecular-level insight into the design principles of ESIPT-assisted TADF emitters.

## Graphical abstract

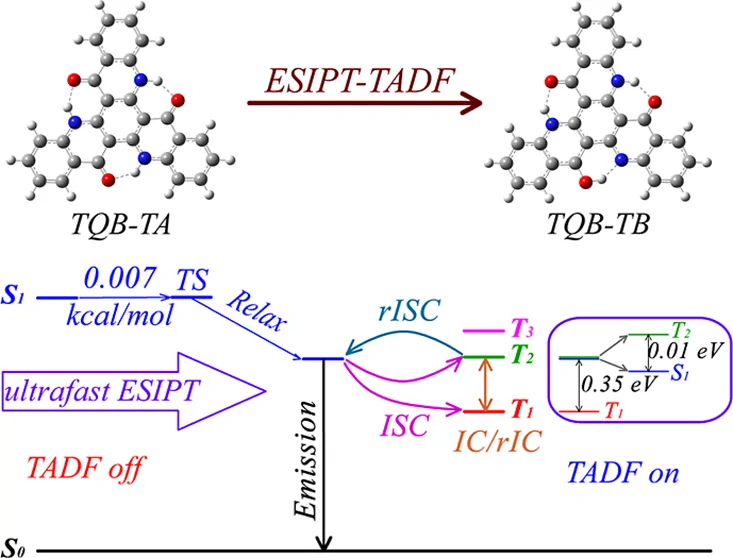

## ■ Introduction

As the third generation of organic light-emitting diode (OLED) emitters, thermally activated delayed fluorescence (TADF) materials enable nearly complete harvesting of both singlet and triplet excitons without the need for heavy-metal components.^1–11^ Since the pioneering report by Adachi and co-workers on the first purely organic TADF emitter in 2011,^1^ tremendous efforts have been devoted to advancing both the fundamental understanding and practical applications of TADF materials.^12–30^ Nevertheless, the vast majority of TADF systems still rely on intra- or intermolecular donor–acceptor (D–A) architectures that exhibit charge-transfer (CT) excited states.^31,32^ The CT character promotes spatial separation of the HOMO and LUMO, thereby reducing the singlet–triplet energy gap (Δ*E*_st_), which is crucial for efficient reverse intersystem crossing (rISC). However, this same orbital separation also weakens the transition dipole moment, resulting in a reduced radiative decay rate constant (*k*_*f*_) due to diminished electron–hole overlap. Consequently, a fundamental trade-off exists in conventional TADF design: achieving a small Δ*E*_st_ often comes at the expense of a high *k*_*f*_, even though both are indispensable for high-performance TADF emitters and devices.^11,33^

In this regard, TADF systems incorporating excited-state intramolecular proton transfer (ESIPT) have emerged as a promising alternative. ESIPT is a well-established photophysical process that has been extensively investigated through both experimental and theoretical studies.^34–41^ It produces large Stokes shifts that effectively suppress self-absorption, enhances molecular rigidity through intramolecular hydrogen bonding to reduce nonradiative decay, and provides unique opportunities for coupling proton transfer with delayed fluorescence. Collectively, these characteristics offer an attractive strategy for improving exciton utilization, enhancing luminescence efficiency, and achieving superior operational stability in TADF materials.^28–30^

Triquinolonobenzene (TQB), a representative excited-state intramolecular proton transfer-induced TADF (ESIPT-TADF) emitter, was first reported by Mamada and co-workers, exhibiting a photoluminescence quantum yield approaching 60% and an external quantum efficiency of 14% in OLED devices.^42^ In the ESIPT product TQB-TB (see Figure 1), the spatial separation of the HOMO and LUMO leads to a smaller singlet–triplet energy gap (Δ*E*_st_) than that of the normal form, TQB-TA. However, subsequent *ab initio* molecular dynamics (AIMD) simulations by Cao and co-workers suggested that this reduced Δ*E*_st_ between the S_1_ and T_1_ states contributes little to triplet harvesting.^43^ Their simulations indicated that the diabatic T_2_ state is populated before the ESIPT reaction occurs, implying that the proton transfer process from ^3^TQB-TA to ^1^TQB-TB, rather than the reduced Δ*E*_st_, plays the dominant role in triplet harvesting. In contrast, transient absorption and time-resolved photoluminescence measurements by Long and co-workers demonstrated that rISC proceeds from the T_2_ to the S_1_ state in the TQB-TB tautomer generated following ultrafast ESIPT.^44^ This mechanistic picture differs fundamentally from that proposed by the AIMD study, in which T_2_ is populated before proton transfer.^43^ Consequently, the detailed TADF mechanism in TQB, particularly the role of ESIPT in triplet harvesting, remains controversial and requires further clarification.

**Figure 1:**
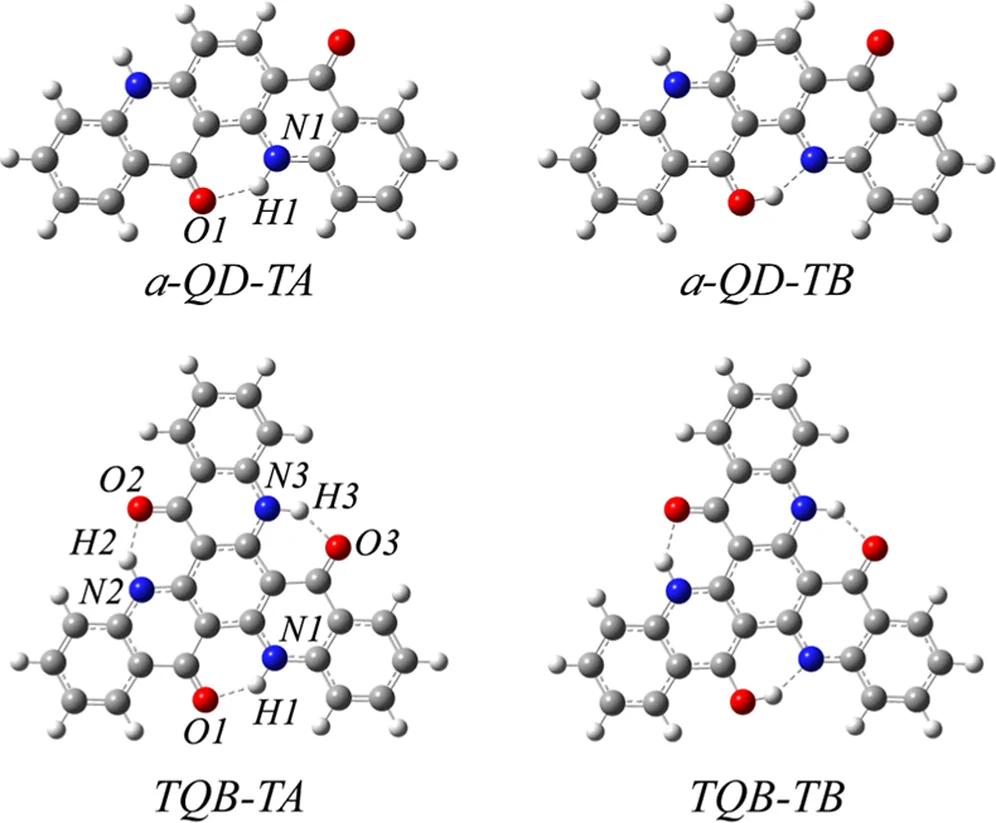
Molecular structures of *a*-QD-TA, *a*-QD-TB, TQB-TA, and TQB-TB with atom labels.

In this work, we employ time-dependent density functional theory (TDDFT) calculations to elucidate the dominant triplet-harvesting pathway in TQB and to clarify the functional role of ESIPT in its TADF process. As a comparative system, we also investigate *a*-QD, which exhibits similar ESIPT characteristics but does not display TADF. By constructing excited-state potential energy profiles and evaluating spin–orbit couplings (SOCs), we demonstrate that intersystem crossing (ISC) from S_1_ to T_1_–T_3_ is effectively suppressed in both the initial TQB-TA form and along the proton-transfer pathway because of the ultrafast ESIPT process and the negligible SOC strengths. We therefore focus on the proton-transfer product (TB) of both systems and evaluate the ISC and rISC rate constants between the S_1_ and T_1_/T_2_ states to identify the dominant triplet-harvesting mechanism in TQB. Furthermore, a comparative analysis of the excited-state photophysical evolution in TQB and *a*-QD reveals the key factor responsible for the markedly enhanced ISC efficiency in TQB-TB, providing new insights into the design principles of high-performance ESIPT-TADF emitters.

## ■ Theoretical Method

All ground-state geometry optimizations were performed using density functional theory (DFT) with the B3LYP exchange–correlation functional.^45^ The triple-ζ valence polarized (TZVP) basis set^46^ was employed throughout this work to provide a balanced description of molecular geometries and electronic structures while maintaining computational efficiency. Excited-state electronic structures were subsequently investigated using time-dependent density functional theory (TDDFT)^47^ at the same level of theory. This computational protocol has been widely applied to the study of excited-state intramolecular proton transfer (ESIPT) and thermally activated delayed fluorescence (TADF) systems, where it provides reliable descriptions of excitation energies, excited-state potential energy surfaces, and the relative ordering of low-lying singlet and triplet states. To mimic the experimental conditions, solvent effects of toluene (dielectric constant, ε = 2.3741) were incorporated using the polarizable continuum model (PCM).^48^ Dispersion interactions were further accounted for using Grimme’s D3 empirical dispersion correction with Becke–Johnson damping (D3BJ),^49^ which improves the description of weak intramolecular interactions and hydrogen-bonding geometries that are important for accurately characterizing the ESIPT process. Frequency calculations were carried out at the same level of theory to verify that all optimized structures correspond to true local minima on the potential energy surface. All DFT and TDDFT calculations were performed using the Gaussian 16 software package.^50^

Because B3LYP is known to be less accurate for excited states with pronounced charge-transfer character, we performed benchmark calculations to assess its reliability. Three additional functionals—PBE0,^51^ CAM-B3LYP,^52^ and ωB97XD^53^—were employed to calculate the absorption and emission energies, which were then compared with the experimental results.^42^ Specifically, the S_0_ → S_1_ vertical excitation energies were calculated at the optimized S_0_ geometries of *a*-QD-TA and TQB-TA, while the S_1_ → S_0_ vertical de-excitation energies were calculated at the optimized S_1_ geometries of *a*-QD-TB and TQB-TB. The results are summarized in Tables S1 and S2 of the Supporting Information. For both excitation and de-excitation energies, the B3LYP results show substantially better agreement with the experimental measurements^42^ than those obtained using the PBE0, CAM-B3LYP, and ωB97XD functionals. In addition, the relative ordering of the S_1_ and T_2_ states depends on the choice of functional. For TQB-TB, B3LYP and PBE0 predict T_2_ to lie slightly above S_1_, whereas CAM-B3LYP and ωB97XD place T_2_ below S_1_. The experimentally determined T_2_-S_1_ energy gap for TQB is approximately 20 meV, with T_2_ lying above S_1_,^44^ which is consistent with the state ordering predicted by B3LYP and PBE0. These results support the reliability of B3LYP for the systems investigated in this work. Cartesian coordinates for all optimized geometries, including 16 local minima on the S_0_, S_1_, T_1_, T_2_, and T_3_ states and two transition states on the S_1_ state, are provided in Table S6 of the Supporting Information.

To elucidate the excited-state relaxation pathways relevant to TADF, spin–orbit coupling (SOC) matrix elements and intersystem crossing (ISC) rate constants (*k*_ISC_) were evaluated at the same B3LYP/TZVP level at 300 K, including both PCM solvation and D3BJ dispersion corrections, using the ORCA (version 5.0.4) program package.^54–56^ SOC matrix elements provide a quantitative measure of the electronic coupling between singlet and triplet states, calculated by the Breit–Pauli SOC operator.^57^ The ISC and the rISC rates between a given initial state *i* and a final state *f* are calculated from Fermi’s Golden rule.^58,59^

1 $$k{\left(\omega \right)}_{if}=\frac{2\pi }{\hbar }{\left|\left\langle \psi_{f}|{Ĥ}_{SO}|\psi_{i}\right\rangle \right|}^{2}\delta \left(E_{i}-E_{f}\right)$$

All normal modes of the initial and final states were explicitly included in the Franck–Condon (FC) and Herzberg–Teller (HT) integrals without truncation. Duschinsky rotations^60,61^ were fully considered, and each electronic state was represented using its own Hessian, thereby accounting for the frequency shifts between the initial and final states. The calculated ISC and the rISC rate constants allow direct assessment of the efficiency of spin-forbidden transitions. Combined with the computed excited-state potential energy profiles, these calculations enable a comprehensive analysis of the competing ESIPT, fluorescence, ISC, and rISC processes, thereby clarifying the dominant triplet-harvesting mechanism in the investigated ESIPT-TADF systems.

To quantify the triplet-harvesting pathway involving the T_1_ and T_2_ states, the T_2_ → T_1_ internal conversion (IC) rate constant was calculated using the FCclasses3 program.^62^ Within the adiabatic Hessian (AH) model, each state is characterized by its own optimized geometry, Hessian, and harmonic frequencies, while the Duschinsky rotation between the corresponding sets of normal modes is fully accounted for. The IC rate is evaluated within the same Fermi golden rule^58,59^ framework used for the ISC rates (eq 1), with the spin–orbit coupling (SOC) replaced by the nonadiabatic coupling (NAC) vector between the two adiabatic states, expressed as

2 $$\Lambda_{if}=\left\langle e_{i}|\nabla_{Q}|e_{f}\right\rangle$$

The T_1_–T_2_ nonadiabatic coupling (NAC) vector was obtained from TD-DFT through numerical differentiation, with T_1_ treated as an open-shell reference state with multiplicity 3 and T_2_ represented by the lowest TD-DFT root. The geometries, Hessians, and energies of both states were calculated at the same B3LYP/TZVP level with PCM solvation. The reverse T_1_–T_2_ rate constant was then determined from the forward rate using detailed balance,

3 $$k_{rIC}=k_{IC}e^{-\Delta E/k_{B}T}$$

with temperature *T* = 300 K.

## ■ Results and Discussion

To elucidate the ESIPT-induced TADF mechanism in TQB, we also investigated angular-quinacridone (*a*-QD), which undergoes a similar excited-state intramolecular proton transfer (ESIPT) process but does not exhibit TADF, as reported by Mamada and co-workers.^42^ Thus, *a*-QD serves as an ideal reference system for identifying the photophysical factors responsible for the distinct TADF behavior of TQB. The optimized structures of the TA (enol) and TB (keto) forms of both TQB and *a*-QD are shown in Figure 1. Geometry optimizations were performed for both the ground (S_0_) and first excited (S_1_) states. It should be noted that the TB forms of both molecules are unstable in the ground state. All S_0_ optimizations initiated from the TB geometries spontaneously relax to the corresponding TA forms, indicating that the keto tautomer is stabilized only upon photoexcitation. All optimized structures retain highly planar geometries with extended π-conjugation, which is favorable for efficient electronic delocalization and excited-state relaxation. The calculated hydrogen-bond lengths in the S_0_ and S_1_ states are summarized in Table 1, providing important structural insights into the ESIPT process. For *a*-QD-TA, photoexcitation significantly strengthens the intramolecular hydrogen bond, as evidenced by the shortening of the N–H···O distance from 1.784 Å in S_0_ to 1.468 Å in S_1_. Such pronounced excited-state hydrogen-bond strengthening lowers the proton-transfer barrier and strongly facilitates ESIPT, a characteristic feature consistently observed in our previous theoretical investigations of ESIPT systems.^38–41^

**Table 1:** Calculated Hydrogen-Bond Lengths (Å) of *a*-QD-TA, *a*-QD-TB, TQB-TA, and TQB-TB in the S_0_ and S_1_ States

species	bond	S_0_	S_1_	species	bond	S_1_
*a*-QD-TA	N1–H1	1.023	1.106	*a*-QD-TB	N1···H1	1.670
	H1···O1	1.784	1.468		H1–O1	1.006
TQB-TA	N1–H1	1.031	1.033	TQB-TB	N1···H1	1.690
	H1···O1	1.707	1.707		H1–O1	0.994
	N2–H2	1.031	1.033		N2–H2	1.024
	H2···O2	1.707	1.709		H2···O2	1.752
	N3–H3	1.031	1.032		N3–H3	1.022
	H3···O3	1.707	1.712		H3···O3	1.767

In contrast, TQB exhibits a markedly different behavior. Owing to its C_3_h-symmetric structure, the ground-state TA form contains three equivalent N–H···O hydrogen bonds (N1–H1···O1, N2–H2···O2, and N3–H3···O3) with essentially identical bond lengths. Upon photoexcitation, however, the symmetry of the hydrogen-bond network is only weakly perturbed. The N1–H1···O1 hydrogen bond remains nearly unchanged, whereas the N2–H2···O2 and N3–H3···O3 hydrogen bonds become slightly longer by 0.002 and 0.005 Å, respectively, indicating a small but discernible weakening. Following the first proton transfer to form the TQB-TB tautomer, the remaining two hydrogen bonds are further elongated in the S_1_ state, suggesting that they become even less favorable for subsequent proton transfer. These structural changes indicate that the initial ESIPT event effectively suppresses additional proton-transfer processes by weakening the remaining hydrogen-bond network. Consequently, sequential double- or triple-proton transfer becomes energetically unfavorable after formation of the first keto tautomer. This finding is fully consistent with the experimental proposal by Mamada and co-workers^42^ that the doubly and triply proton-transferred species (TQB-TC and TQB-TD) are unlikely to be generated under photoexcitation. More importantly, the calculations demonstrate that the excited-state dynamics of TQB are dominated by a single ESIPT event, thereby considerably simplifying the subsequent analysis of the TADF mechanism by restricting the relevant photophysical processes to the TQB-TB tautomer.

The calculated vertical absorption (S_0_ → S_1_) and emission (S_1_ → S_0_) wavelengths, together with the corresponding dominant orbital transitions, are summarized in Table 2 and compared with the experimental spectra reported by Mamada and co-workers.^42^ Both *a*-QD and TQB exhibit exceptionally large Stokes shifts, which are characteristic signatures of excited-state intramolecular proton transfer (ESIPT). The calculated absorption maxima of the TA forms are 398.47 and 364.85 nm for *a*-QD and TQB, respectively, whereas the corresponding emissions from the TB tautomers are predicted at 661.28 and 534.29 nm. The calculated spectra reproduce the experimental trend well, confirming that photoexcitation initially populates the enol (TA) form, while fluorescence originates exclusively from the proton-transferred keto (TB) tautomer. Notably, the complete absence of “normal” fluorescence from the TA form in the experimental spectra implies that ESIPT proceeds much faster than radiative decay, consistent with an essentially barrierless proton-transfer process in the S_1_ state. Overall, the excellent agreement between the calculated and experimental spectra validates the reliability of the present TDDFT methodology for describing the excited-state electronic structures of both systems.

**Table 2:** Calculated Absorption (S_0_ → S_1_) and Emission (S_1_ → S_0_) Wavelengths (nm) and the Corresponding Orbital Transitions (OTs) of *a*-QD-TA, *a*-QD-TB, TQB-TA, and TQB-TB in Toluene with the Corresponding Experiment Values^42^

	absorption (S_0_ → S_1_)			emission (S_1_ → S_0_)		
species	wavelength	OT	exp.^a^	wavelength	OT	exp.^a^
*a*-QD-TA	398.47	H → L (97.0%)	400–410	467.20	H → L (98.7%)	—
*a*-QD-TB	—	—	—	661.28	H → L (99.3%)	650–660
TQB-TA	364.85	H(1) → L(1)	360–370	376.28	H(1) → L(1)	—
		(47.8%)			(47.1%)	
		H(2) → L(2)			H(2) → L(2)	
		(47.7%)			(45.5%)	
TQB-TB	—	—	—	534.29	H→L (98.8%)	510–520

a Ref 42.

An intriguing feature of TQB-TA is the presence of doubly degenerate frontier molecular orbitals, denoted as HOMO(1), HOMO(2), LUMO(1), and LUMO(2). Consequently, the S_0_ ↔ S_1_ transition is composed of two nearly equivalent π–π* excitations, HOMO(1) → LUMO(1) and HOMO(2) → LUMO(2), reflecting the high symmetry of the molecule. The frontier molecular orbitals of both *a*-QD-TA and TQB-TA are illustrated in Figure 2, demonstrating that the lowest excited state possesses predominantly ππ* character in both compounds. However, frontier orbitals alone cannot fully describe the redistribution of electron density following excitation, particularly for TQB because of its orbital degeneracy. To obtain a more intuitive picture of the photoinduced charge redistribution, charge density difference (CDD) analyses were performed using the Multiwfn program.^63,64^

**Figure 2:**
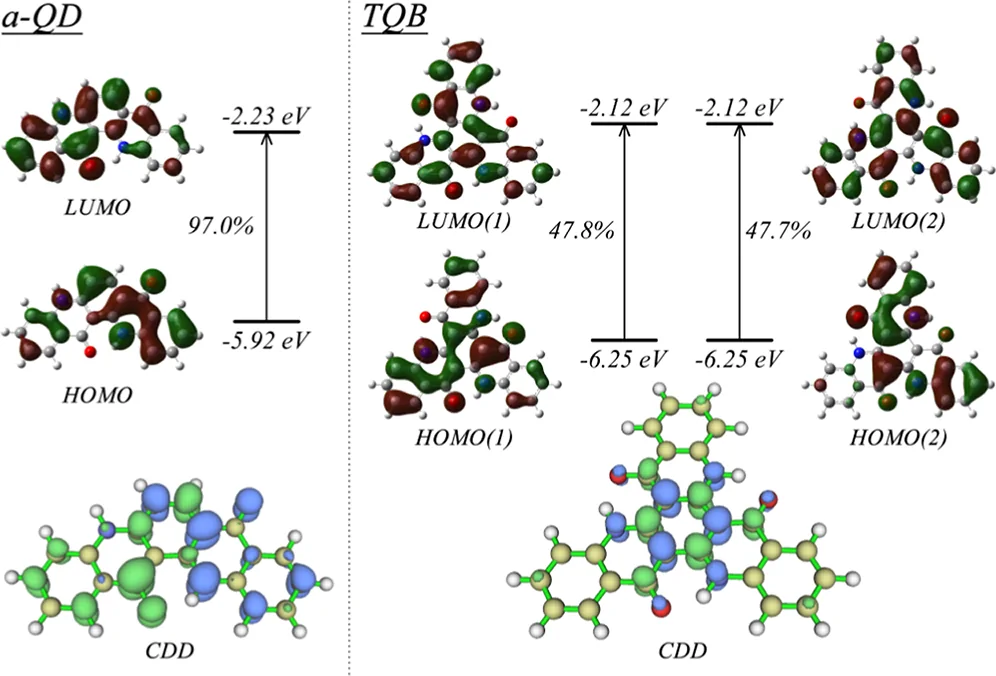
Frontier molecular orbitals (MOs) contributing predominantly to the S_1_ wave function of *a*-QD-TA and TQB-TA with the corresponding charge density difference (CDD) maps. Green and blue isosurfaces in the CDD maps represent electron accumulation and hole (electron depletion) regions, respectively.

The CDD map of *a*-QD-TA clearly reveals electron accumulation around the carbonyl oxygen, the proton acceptor, accompanied by electron depletion at the N–H proton donor. This redistribution enhances the basicity of the oxygen atom while weakening the N–H bond, thereby strengthening the intramolecular hydrogen bond and facilitating proton transfer in the excited state. The electronic redistribution is therefore fully consistent with the pronounced shortening of the hydrogen bond discussed above. In contrast, TQB-TA exhibits a substantially different charge redistribution pattern. Electron density decreases simultaneously around both the proton donor and acceptor sites, indicating that excitation does not directly reinforce the proton-transfer coordinate. Instead, the electronic excitation is delocalized over the highly symmetric π-conjugated framework, suggesting that the driving force for ESIPT in TQB originates from a more subtle balance between electronic delocalization and nuclear relaxation rather than simple donor-to-acceptor charge redistribution. This observation explains why the excited-state hydrogen-bond strengthening in TQB is much weaker than that in *a*-QD.

To further elucidate the proton-transfer mechanism, the excited-state potential energy profiles along the proton-transfer coordinate were constructed for both systems, as shown in Figure 3. The reaction pathways include the initially excited local minimum (LE), the relaxed TA structure, the transition state (TS), and the proton-transferred TB product. The calculated ESIPT barrier of TQB is 0.079 kcal·mol^–1^, which is more than 1 order of magnitude larger than that of *a*-QD (0.007 kcal·mol^–1^). Although this result confirms that proton transfer in TQB is intrinsically less favorable than in *a*-QD, consistent with the CDD analysis, both barriers are sufficiently small to render the ESIPT process effectively barrierless. These calculations therefore explain why both compounds exhibit ultrafast ESIPT, despite their distinct electronic redistribution mechanisms.

**Figure 3:**
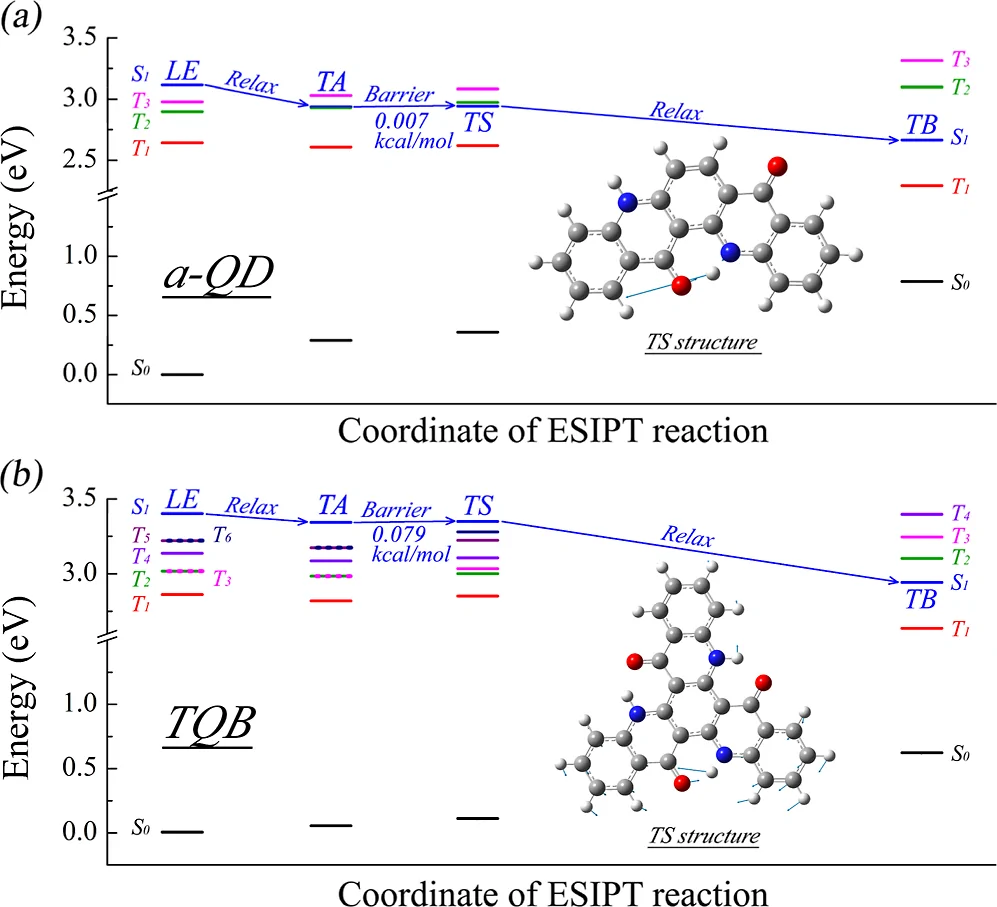
Potential energy profiles along the ESIPT coordinate on the S_1_ state and relevant triplet (T_n_) potential energy surfaces for (a) *a*-QD and (b) TQB. The S_1_ Transition-state (TS) structures were fully optimized, whereas the triplet-state energies were obtained from single-point calculations at the corresponding S_1_ geometries.

The present results are also consistent with the previous *ab initio* molecular dynamics simulations of Cao and co-workers,^43^ which predicted that ESIPT in TQB occurs within approximately 20 fs after photoexcitation. Such a time scale is considerably shorter than those of fluorescence and typical intersystem crossing processes, indicating that the initially excited TA form exists only transiently before converting into the TB tautomer. Consequently, the proton-transferred TB species should be regarded as the primary excited-state intermediate responsible for subsequent photophysical processes.

Following the mechanism proposed by Cao and co-workers,^43^ the ESIPT process itself is not considered to directly enhance triplet harvesting but instead provides access to multiple triplet states during proton transfer. To examine this possibility, we calculated the energies of the low-lying triplet states along the ESIPT coordinate. As shown in Figure 3, substantially more triplet states intersect the S_1_ potential energy profile in TQB than in *a*-QD, suggesting that TQB possesses a greater number of potential ISC channels. At first glance, this feature appears to rationalize why TQB exhibits TADF, whereas *a*-QD does not. However, the mere existence of energetic crossings does not guarantee efficient spin conversion. Because ESIPT is completed within only ∼20 fs, any ISC occurring before proton transfer must compete with an extremely rapid nuclear motion. Whether spin conversion can proceed efficiently on such an ultrafast time scale depends critically on the magnitude of the spin–orbit coupling and the corresponding ISC rate constants. This issue is therefore examined quantitatively in the following discussion.

To evaluate whether intersystem crossing (ISC) can compete with the ultrafast ESIPT process, we investigated the S_1_ → T_1_–T_3_ spin conversion by optimizing a series of constrained S_1_ geometries along the proton-transfer coordinate (defined by the N1–H1 bond length) for both molecules. The resulting excited-state energy profiles are shown in Figure 4 together with the corresponding spin–orbit coupling (SOC) matrix elements calculated using the ORCA package.^54–56^ The triplet states remain well separated and retain their energy ordering throughout the entire scan, with no avoided crossings observed in Tables S4 and S5 of Supporting Information. Following the entire ESIPT scan, we calculated the energy gaps between adjacent triplet states as functions of the N1–H1 bond length for both a-QD and TQB as shown in Figure S1 of the Supporting Information, where the E­(T_3_)–E­(T_2_) gap for TQB reaches a minimum of approximately 1.5 meV near the enol side, where the two states are nearly degenerate, and then increases monotonically as proton transfer proceeds. Thus, no root flipping occurs, and each adiabatic state can be tracked continuously and unambiguously throughout the scan. The same conclusion applies to the a-QD system. For *a*-QD, the SOC matrix elements between S_1_ and the three lowest triplet states remain essentially zero throughout the entire proton-transfer pathway, indicating that ISC is negligible before completion of ESIPT. This result is fully consistent with the experimentally observed absence of TADF in *a*-QD. Although TQB exhibits slightly larger SOC fluctuations along the reaction coordinate, all calculated values remain below ∼0.6 cm^–1^. Because the relevant frontier orbitals are of π character in Figure S2 and Table S3 of the Supporting Information, the S_1_, T_1_, T_2_, and T_3_ states all possess predominantly ππ* character. Accordingly, the S_1_ → T_n_ transitions involve coupling between states of the same electronic character (ππ* → ππ*), which is disfavored by El-Sayed’s rule and consequently leads to weak spin–orbit coupling (SOC). Such weak spin–orbit interactions are insufficient to promote spin conversion on a time scale comparable to the ∼20 fs ESIPT process. Therefore, the existence of multiple S_1_/T_n_ crossings along the proton-transfer pathway, as shown in Figure 4b, does not necessarily imply efficient ISC.

**Figure 4:**
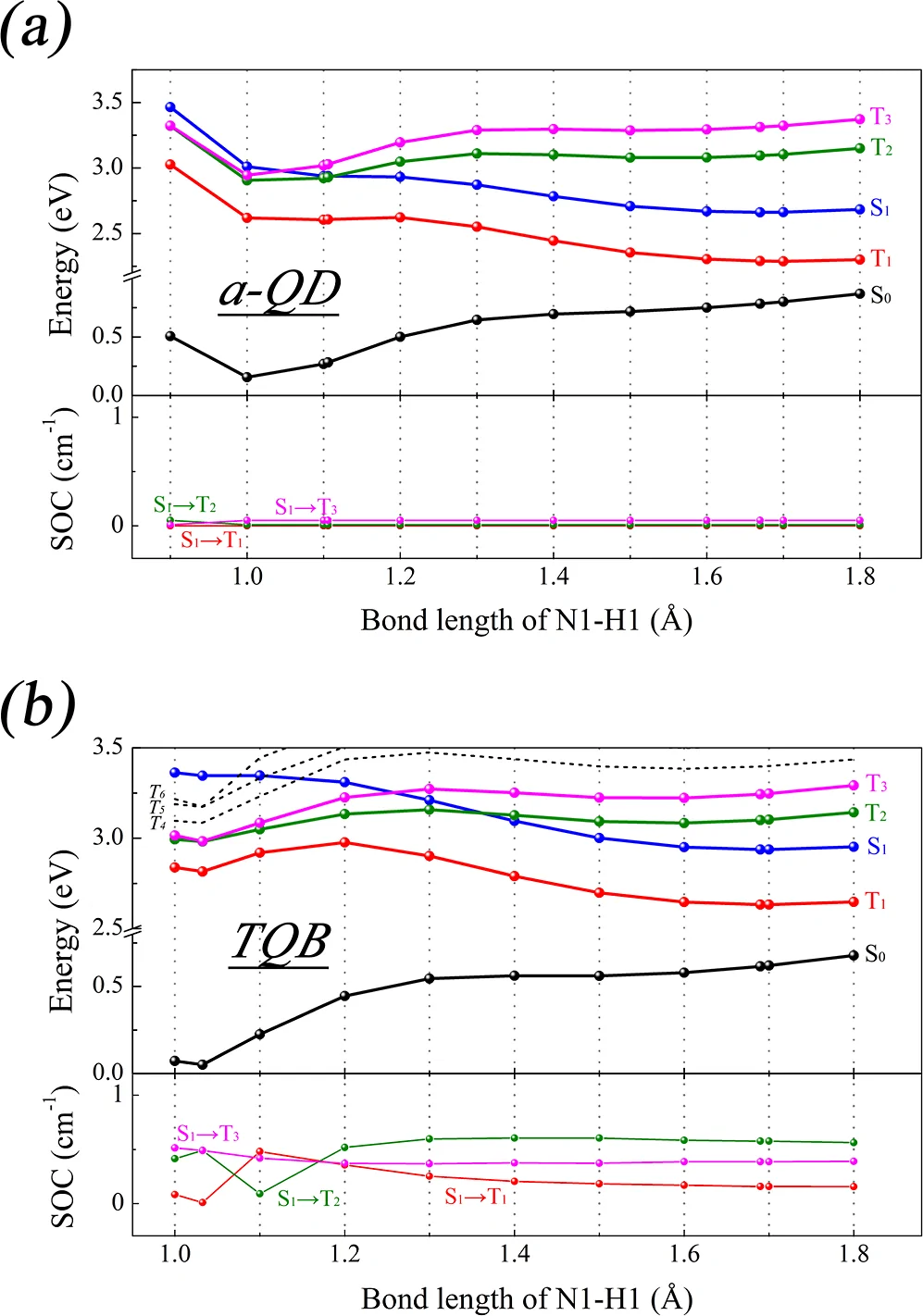
Potential energy profiles of the S_1_, T_1_, T_2_, and T_3_ states along the ESIPT coordinate for (a) *a*-QD and (b) TQB, obtained from constrained S_1_-state geometry optimizations with fixed N1–H1 bond distances using Gaussian 16. The corresponding spin–orbital coupling (SOC) matrix elements for S_1_ → T_n_ (*n* = 1–3) transitions, calculated with ORCA, are also shown. The detailed data shown in the figure are listed in Tables S4 and S5 (SI).

To quantify this conclusion, we calculated the ISC rate constants along the ESIPT pathway. Among all channels, the S_1_ → T_2_ transition of TQB-TA exhibits the largest SOC and has previously been proposed as the dominant ISC pathway based on both AIMD simulations^43^ and time-resolved spectroscopic measurements.^44^ Within the Herzberg–Teller (HT) formalism, the corresponding ISC rate constant is calculated to be 1.50 × 10^9^ s^–1^, corresponding to an ISC lifetime of 0.7 ns. This time scale is approximately 5 orders of magnitude slower than the ESIPT process (∼20 fs), demonstrating that proton transfer is completed long before any appreciable spin conversion can occur. Consequently, the initially excited TA form has essentially no opportunity to undergo ISC prior to ESIPT. Moreover, even under the hypothetical assumption that a small fraction of the excited-state population undergoes ISC before proton transfer, the resulting triplet population would remain confined to the TA potential energy surface because of the substantial triplet-state barrier along the proton-transfer coordinate (Figure 4b). Such a process would therefore neither generate the emissive TB tautomer nor contribute to the experimentally observed delayed fluorescence. These results collectively exclude the possibility that triplet harvesting occurs before or during ESIPT, providing strong theoretical evidence against the previously proposed AIMD mechanism involving the ^3^TQB-TA → ^1^TQB-TB pathway.^43^ Instead, our calculations indicate that ESIPT must precede any efficient spin conversion.

Based on the above analysis, the only viable pathway for triplet harvesting is ISC occurring after completion of ESIPT, namely, in the proton-transferred TB tautomer. Accordingly, the ISC and reverse ISC (rISC) rate constants between S_1_ and T_1_/T_2_ were calculated for both *a*-QD-TB and TQB-TB within the HT formalism and are summarized in Table 3. Because the TB tautomers are unstable in the ground state and cannot be optimized as S_0_ minima, conventional Franck–Condon (FC) simulations of fluorescence are not applicable. Instead, the fluorescence rate constants were estimated using the Einstein spontaneous–emission relation, *k*_*f*_ = *f* × *E*_if_^2^/1.499, where *f* is the oscillator strength and *E*_if_ is the S_1_–S_0_ transition energy. The complete excited-state kinetic schemes, including fluorescence, ISC, and rISC together with their calculated rate constants, are summarized in Figure 5.

**Table 3:** Calculated ISC and Spontaneous Emission Rate Constants for *a*-QD-TB and TQB-TB with Franck–Condon (FC) and Herzberg–Teller (HT) Contributions to ISC Rates

state	*a*-QD-TB				TQB-TB			
	*k*_ISC_ (s^–1^)	contribution		*k*_*f*_ (s^–1^)	*k*_ISC_ (s^–1^)	contribution		*k*_*f*_ (s^–1^)
		FC	HT			FC	HT	
S_1_ → S_0_	—	—	—	8.71 × 10^7^	—	—	—	4.14 × 10^7^
S_1_ → T_1_	1.63 × 10^7^	0.00%	100.00%	—	1.10 × 10^8^	3.08%	96.92%	—
S_1_ → T_2_	4.37 × 10^4^	0.01%	99.99%	—	1.37 × 10^8^	41.82%	58.18%	—
T_1_ → S_1_	2.89 × 10°	0.00%	100.00%	—	1.18 × 10^2^	1.81%	98.19%	—
T_2_ → S_1_	7.31 × 10^8^	0.00%	100.00%	—	9.70 × 10^7^	26.10%	73.90%	—

**Figure 5:**
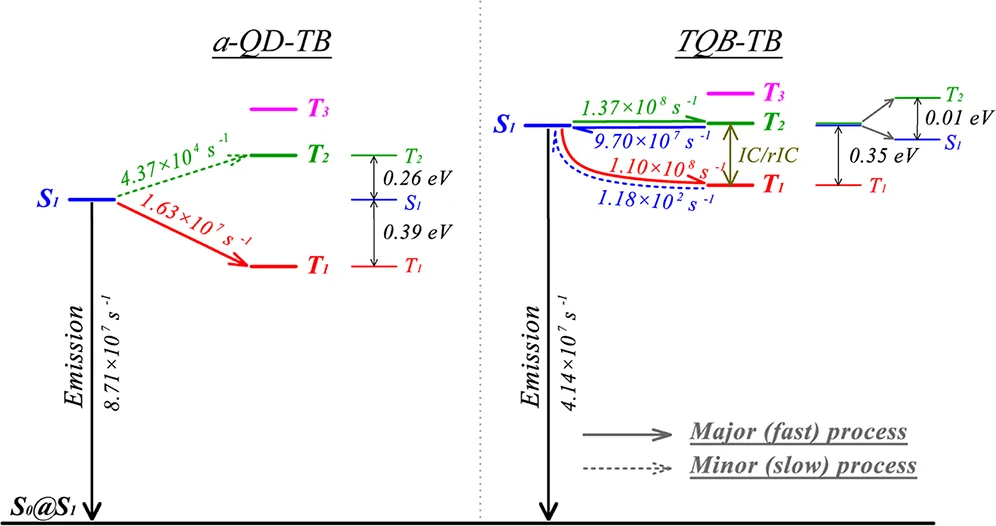
Schematic illustration of key photophysical pathways, including fluorescence, intersystem crossing (ISC), and reverse intersystem crossing (rISC) with the corresponding rate constants for *a*-QD-TB and TQB-TB.

For *a*-QD-TB, the fastest ISC pathway is S_1_ → T_1_ with a rate constant of 1.63 × 10^7^ s^–1^. However, the calculated fluorescence rate constant (8.71 × 10^7^ s^–1^) is more than five times larger, indicating that radiative decay dominates the excited-state deactivation. Although a small fraction of molecules can populate the triplet manifold through ISC, the reverse process is essentially forbidden because of the large S_1_–T_1_ energy gap (0.39 eV), resulting in an extremely small rISC rate constant of only 2.89 s^–1^. Consequently, the triplet excitons cannot be recycled back to the emissive singlet state. On the other hand, although the T_2_ → S_1_ reverse intersystem crossing (rISC) is very fast, with a rate constant of 7.31 × 10^8^ s^–1^, the corresponding S_1_ → T_2_ intersystem crossing (ISC) is extremely slow, with a rate constant of only 4.37 × 10^4^ s^–1^. Consequently, the T_2_ state of *a*-QD-TB is scarcely populated, leaving the rapid reverse process largely unused. Thus, neither triplet pathway can sustain a complete ISC–rISC recycling cycle, providing a quantitative explanation for the absence of TADF in *a*-QD. The situation changes dramatically for TQB-TB. The ISC rate constants for the S_1_ → T_1_ and S_1_ → T_2_ channels are 1.10 × 10^8^ and 1.37 × 10^8^ s^–1^, respectively, both exceeding the fluorescence rate constant. Therefore, after ESIPT, the dominant relaxation pathway is ISC rather than prompt fluorescence (PF), leading to an efficient population of the triplet manifold. This efficient ISC pathway competes with prompt fluorescence (PF) from the S_1_ state and consequently limits the prompt fluorescence quantum yield (PFQY) of TQB. Mamada and co-workers reported a PFQY of 46% for TQB-doped films,^42^ which is lower than that of many high-performance D–A­(–D) TADF materials.^65,66^ Using the ISC and stimulated emission (SE) rate constants listed in Table 3, the prompt fluorescence lifetime can be estimated as τ_PF_ = (*k*_*f*_ + *k*_S1 → T1_ + *k*_S1 → T2_)^−1^ = 3.5 ns. This value is in reasonable agreement with the experimental lifetime of 6.5 ns measured for TQB-doped films by Long and co-workers.^44^ More importantly, the minimal S_1_–T_2_ energy gap (0.01 eV) gives rise to an exceptionally fast rISC process (9.70 × 10^7^ s^–1^), allowing triplet excitons to be thermally upconverted to S_1_ with high efficiency. Although direct T_1_ → S_1_ rISC remains inefficient because of the much larger energy gap, recent time-resolved spectroscopic measurements by Long and co-workers^44^ demonstrated that T_1_ is thermally repopulated to T_2_ through reverse internal conversion (rIC) with an energy gap of 180 meV. To complete the description of the triplet-harvesting mechanism, the T_2_ → T_1_ internal conversion (IC) rate constant was estimated using the FCclasses3 program^62^ and the T_1_–T_2_ nonadiabatic coupling obtained from TD-DFT. The calculated rate, *k*_IC(T2→T1)_ ≈ 1.29 × 10^13^ s^–1^, indicates that T_2_ decays to T_1_ on the femtosecond time scale. The reverse T_1_ → T_2_ internal conversion (rIC) rate constant was estimated from detailed balance (eq 3) to be ≈ 9.88 × 10^6^ s^–1^, using an adiabatic gap of 0.36 eV. Thus, the T_1_ → T_2_ rIC is substantially slower than the T_2_ → T_1_ IC, allowing T_1_ to act as a long-lived dark trap for triplet excitons. This provides a rationale for the delayed fluorescence (DF) observed only after approximately 100 ns and its decay on the millisecond time scale.^44^ Consequently, both T_1_-and T_2_-derived triplet excitons ultimately return to the emissive S_1_ state through the T_2_ intermediate, establishing the complete triplet-harvesting mechanism responsible for TADF in TQB.

An additional insight from the present calculations is the critical role of Herzberg–Teller vibronic coupling (HTVC). This vibronic mechanism was recently highlighted by Lin and co-workers,^67,68^ who demonstrated its important role in determining the ISC and rISC rates of TADF emitters and further identified second-order spin–vibronic coupling (SVC) as a distinct mechanism in systems with small singlet–triplet energy gaps. As summarized in Table 3, the HT contribution accounts for nearly the entire ISC rate in most spin-conversion channels. Even for the two most efficient processes, S_1_ → T_2_ and T_2_ → S_1_, where the Franck–Condon contribution becomes appreciable (41.82% and 26.10%, respectively), the HT term remains indispensable for reproducing the overall transition rates. These results demonstrate that vibronic coupling, rather than spin–orbit coupling alone, governs the spin-conversion kinetics in weak-SOC ESIPT systems such as TQB. In contrast, conventional donor–acceptor TADF emitters with intrinsically stronger SOC generally rely predominantly on Franck–Condon coupling to facilitate ISC and rISC. The present study therefore identifies a fundamentally different triplet-harvesting mechanism for ESIPT-TADF materials, in which ultrafast proton transfer first generates the TB tautomer, followed by HT-assisted ISC/rISC within the proton-transferred structure.

## ■ Conclusions

The calculated TD-DFT potential energy surfaces of the S_0_, S_1_, T_1_, and T_2_ states, together with the spin–orbit coupling (SOC) analysis, reveal a comprehensive intersystem crossing (ISC) network that underlies the delayed fluorescence mechanism of the ESIPT-TADF emitter TQB, with *a*-QD serving as a non-TADF reference system. The proposed photophysical mechanism is summarized schematically in Figure 5. Upon photoexcitation, the molecule is promoted vertically from the S_0_ minimum to the Franck–Condon region of the S_1_ state, where it undergoes ultrafast structural relaxation along the S_1_ potential energy surface. The excited-state evolution is dominated by a barrierless ESIPT process, leading directly to the keto tautomer TQB-TB. This prediction is in excellent agreement with previous AIMD simulations, which demonstrated that proton transfer in TQB is completed on an ultrafast time scale of approximately 20 fs.^43^ The ESIPT process not only stabilizes the excited-state keto structure but also dramatically reorganizes the excited-state electronic structure, thereby activating efficient spin-conversion channels. In TQB-TB, the calculated ISC rate constants for the S_1_ → T_1_ and S_1_ → T_2_ transitions reach 1.10 × 10^8^ and 1.37 × 10^8^ s^–1^, respectively. More importantly, the nearly degenerate S_1_ and T_2_ states, separated by only 0.01 eV, facilitate an exceptionally efficient reverse ISC (T_2_ → S_1_), rapidly repopulating the emissive singlet state. These findings establish the sequential S_1_ → T_2_ → S_1_ cycle as the dominant triplet-harvesting pathway responsible for the high TADF efficiency of TQB.

In addition to this primary mechanism, a secondary triplet-harvesting pathway arises through thermally activated reverse internal conversion (rIC) from T_1_ to T_2_, as proposed by Long and co-workers.^44^ Population transferred from T_1_ can therefore be recycled through the T_1_ → T_2_ → S_1_ sequence, providing an additional route for delayed fluorescence. Analysis of the vibronically resolved ISC rate constants further demonstrates that the HT mechanism makes the dominant contribution to both ISC and reverse ISC processes, highlighting the critical role of vibronic coupling in promoting spin-forbidden transitions. Nevertheless, the FC contribution remains far from negligible, accounting for 41.82% of the S_1_ → T_2_ ISC rate and 26.10% of the T_2_ → S_1_ reverse ISC rate. Since these two transitions constitute the key triplet-harvesting channels in TQB-TB, the sizable FC contribution indicates that both FC and HT mechanisms cooperate to achieve highly efficient spin conversion, with the HT effect providing the primary enhancement while the FC term remains indispensable for realizing the remarkable TADF performance of TQB.

Overall, the TD-DFT calculations reveal that the delayed fluorescence mechanism of TQB is governed by a multidimensional ISC network involving the S_1_, T_1_, and T_2_ states of the proton-transferred keto tautomer. Within this network, the T_2_ state acts as the key intermediate that mediates efficient triplet harvesting by enabling rapid reverse ISC to the emissive S_1_ state, while the T_1_ state contributes indirectly through thermally activated rIC to T_2_. These results demonstrate that the interplay between the coupled singlet–triplet potential energy surfaces and vibronic coupling is crucial for controlling the spin-conversion dynamics and delayed fluorescence in ESIPT-TADF systems. The present mechanistic picture provides valuable molecular-level insights into the fundamental design principles for developing highly efficient ESIPT-assisted TADF emitters.

## Supplementary Material



## Notes

The Supporting Information contains additional tables and figures with Cartesian coordinates for all optimized geometries and the corresponding frequencies, including 16 local minima on the S_0_, S_1_, T_1_, T_2_, and T_3_ states and two transition states on the S_1_ state (PDF)
