# Supplementary material for: Excited-State Intramolecular Proton Transfer: A TD-DFT Study of the Delayed Fluorescence Mechanism in Triquinolonobenzene
Source: J Phys Chem A. 2026 Oct 27;130(44):8740–9. doi: 10.1021/acs.jpca.6c04402 (PMC13746801; doi:10.1021/acs.jpca.6c04402)
Supplement: Supplementary file 1 [file jp6c04402_si_001.pdf]

Supporting information for

Excited-State Intramolecular Proton Transfer: A TD-DFT Study of the Delayed Fluorescence Mechanism in Triquinolonobenzene

Yuhui Liu,[a] Mai-Ning Li,[a] Si-Ying Lu,[a] Yajing Peng,[a] Yu Zhao,*,[a] Jun Gao,[b] and Chaoyuan Zhu*,[bc]

[a] *College of Physical Science and Technology, Bohai University, Jinzhou 121013, China*

[b] *Hubei Key Laboratory of Agricultural Bioinformatics, College of Informatics, Huazhong Agricultural University, Wuhan, 430070, P. R. China*

[c] *Department of Applied Chemistry, National Yang Ming Chiao-Tung University, Hsinchu 30010, Taiwan*

* Corresponding author: E-mail: zhaoyu@bhu.edu.cn; cyzhu@mail.nctu.edu.tw

Table S1. The vertical excitation energies from S_0 to S_1, T_1, T_2 and T_3 state calculated at minimum of S_0-state of a-QD-TA and TQB-TA, respectively with the B3LYP, PBE0, CAM-B3LYP, ωB97XD functionals. The experimental absorption band[42] related to the vertical excitation energy of the $S_0 \rightarrow S_1$ transition is compared.

Functionals	a-QD-TA				TQB-TA			
	$S_0 \rightarrow S_1$	$S_0 \rightarrow T_1$	$S_0 \rightarrow T_2$	$S_0 \rightarrow T_3$	$S_0 \rightarrow S_1$	$S_0 \rightarrow T_1$	$S_0 \rightarrow T_2$	$S_0 \rightarrow T_3$
B3LYP	398.5	469.7	428.6	416.5	364.9	433.7	411.7	411.7
PBE0	382.9	463.5	423.4	414.3	352.1	434.1	407.0	407.0
CAM-B3LYP	343.0	435.7	399.9	391.7	320.6	415.8	383.2	383.2
ωB97XD	338.9	428.3	387.6	385.3	317.9	406.2	375.5	375.5
Exp.[a]	400-410				360-370			

[a]Ref.42

Table S2. The vertical deexcitation energies from S_1, T_1, T_2 and T_3 to S_0 calculated at minimum of S_1-state of a-QD-TB and TQB-TB, respectively with the B3LYP, PBE0, CAM-B3LYP, ωB97XD functionals. The experimental emission band[42] related to the vertical deexcitation energy of the $S_1 \rightarrow S_0$ transition is compared.

Functionals	*a*-QD-TB				TQB-TB			
	$S_1 \rightarrow S_0$	$T_1 \rightarrow S_0$	$T_2 \rightarrow S_0$	$T_3 \rightarrow S_0$	$S_1 \rightarrow S_0$	$T_1 \rightarrow S_0$	$T_2 \rightarrow S_0$	$T_3 \rightarrow S_0$
B3LYP	661.3	823.5	536.6	490.4	534.3	614.4	498.9	471.7
PBE0	623.6	782.6	538.6	483.3	503.7	595.0	497.9	467.4
CAM-B3LYP	509.2	631.8	515.5	449.2	413.8	527.8	462.0	439.1
ωB97XD	489.8	601.2	498.1	439.0	401.0	503.9	450.2	427.9
Exp.[a]	650-660				510-520			

[a]Ref.42

Table S3. The orbital transfer (OT) and the corresponding contributions related to S_1, T_1, T_2 and T_3 states of a-QD-TA and TQB-TA, respectively.

State	a-QD-TA		TQB-TA	
	OT	contribution	OT	contribution
S_1	81→82	97.0%	111→112	47.8%
			110→113	47.7%
T_1	81→82	75.6%	110→112	39.1%
			111→113	39.1%
T_2	81→83	77.0%	110→112	18.6%
			111→113	18.6%
			111→114	16.8%
			110→114	14.3%
T_3	80→82	75.1%	110→113	18.6%
			111→112	18.5%
			110→114	16.8%
			111→114	14.3%

Table S4. The energies of different electronic states and the corresponding SOC data along the bond length of N1-H1 in *a*-QD.

Bond length of N1–H1 (Å)	Energies (eV)					SOC (cm^{-1})		
	S_0	S_1	T_1	T_2	T_3	$S_1 \rightarrow T_1$	$S_1 \rightarrow T_2$	$S_1 \rightarrow T_3$
0.9	0.50719	3.46559	3.02639	3.32069	3.32289	0	0.05	0.01
1.0	0.15597	3.01037	2.61997	2.90497	2.94497	0	0.01	0.05
1.1	0.2697	2.9383	2.6048	2.922	3.0186	0	0.01	0.05
1.10578	0.28227	2.93817	2.60727	2.92877	3.02857	0	0.01	0.05
1.2	0.50092	2.93162	2.62262	3.04852	3.19562	0	0.01	0.05
1.3	0.64672	2.87222	2.55192	3.10992	3.28922	0	0.01	0.05
1.4	0.69553	2.78393	2.44633	3.10063	3.29713	0	0.01	0.05
1.5	0.71695	2.70855	2.35575	3.07955	3.28645	0	0.01	0.05
1.6	0.75013	2.66933	2.30423	3.07983	3.29373	0	0.01	0.05
1.66972	0.78416	2.66206	2.28976	3.09486	3.31216	0	0.01	0.05
1.7	0.80129	2.66339	2.28809	3.10429	3.32309	0	0.01	0.05
1.8	0.86893	2.68473	2.30093	3.14983	3.37203	0	0.01	0.05

Table S5. The energies of different electronic states and the corresponding SOC data along the bond length of N1-H1 in TQB.

Bond length of N1-H1 (Å)	Energies (eV)					SOC (cm^{-1})		
	S_0	S_1	T_1	T_2	T_3	$S_1 \rightarrow T_1$	$S_1 \rightarrow T_2$	$S_1 \rightarrow T_3$
1.0	0.07175	3.36205	2.83805	2.99345	3.01505	0.08307	0.41605	0.5143
1.03255	0.05037	3.34557	2.81517	2.98187	2.98337	0.01	0.49031	0.49031
1.1	0.2249	3.3465	2.9198	3.0492	3.0843	0.48166	0.0911	0.42071
1.2	0.44422	3.31012	2.97722	3.13342	3.22542	0.35819	0.51933	0.37135
1.3	0.54474	3.21024	2.90144	3.15734	3.27174	0.25318	0.59666	0.36892
1.4	0.5616	3.0947	2.7904	3.1265	3.2517	0.20469	0.60415	0.37656
1.5	0.56105	3.00155	2.69805	3.09175	3.22445	0.18276	0.60415	0.3735
1.6	0.5794	2.9507	2.647	3.0838	3.2225	0.16882	0.58464	0.38756
1.68986	0.61478	2.93698	2.63288	3.09988	3.24328	0.15843	0.57723	0.38756
1.7	0.61974	2.93704	2.63284	3.10274	3.24674	0.15843	0.57723	0.38756
1.8	0.67779	2.95259	2.64809	3.14319	3.29179	0.15556	0.56303	0.39064

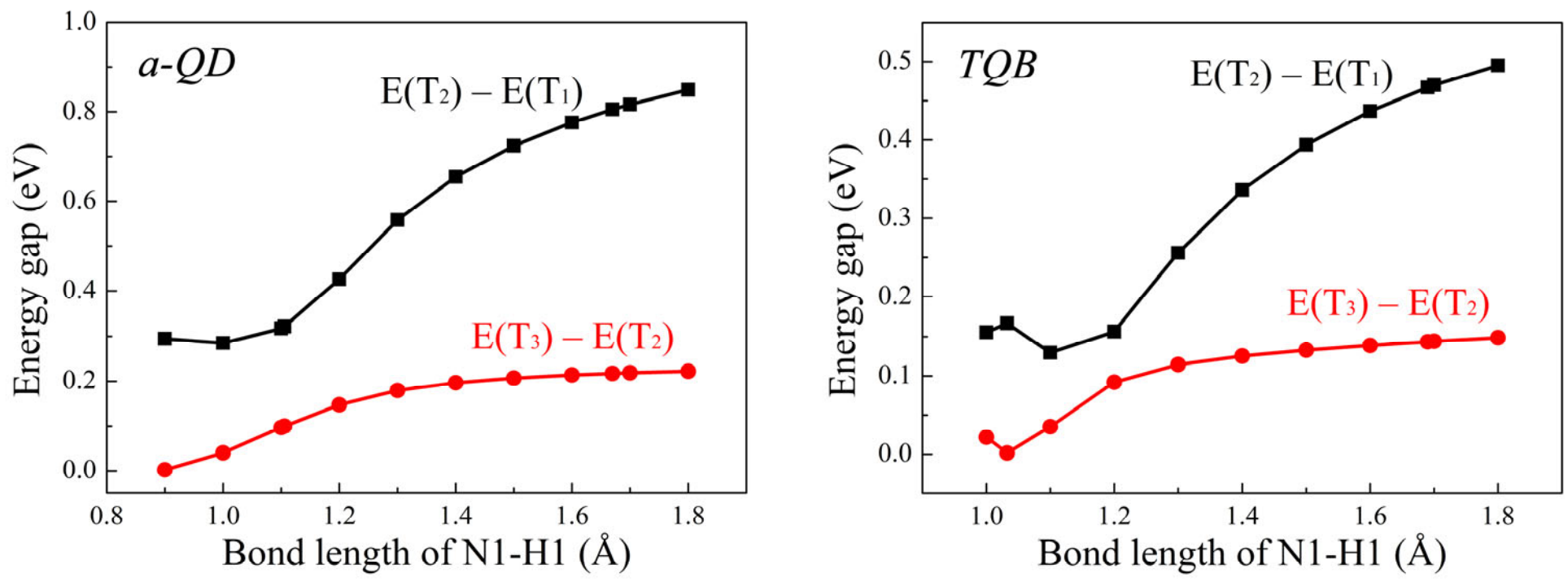

Figure S1. Scanned potential energies of $E(T_2)-E(T_1)$ and $E(T_3)-E(T_2)$ along the ESIPT coordinate (N1-H1 bond length) for a-QD and TQB.

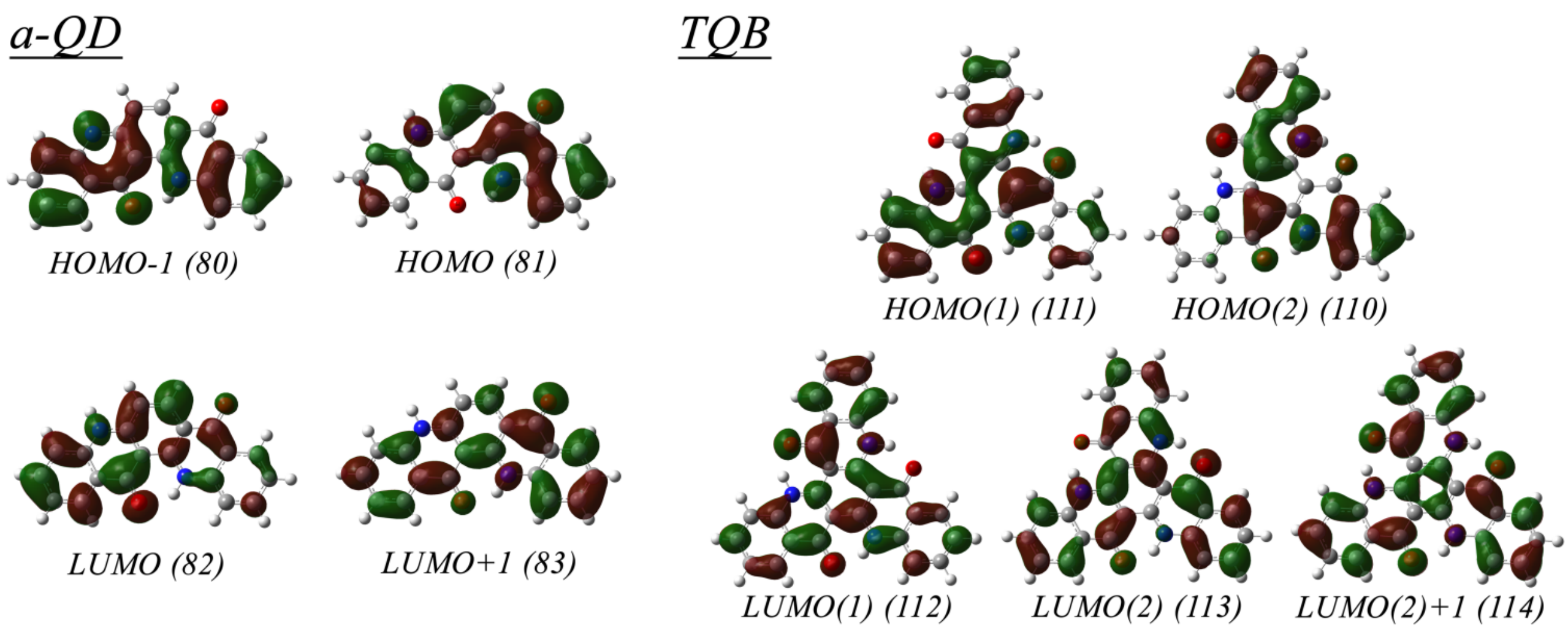

Figure S2. Frontier molecular orbitals (MOs) contributing predominantly to the S_1, T_1, T_2 and T_3 wavefunctions of a-QD-TA and TQB-TA.

Table S6 Cartesian coordinates for optimized geometries and frequencies at the corresponding geometries**.**

S6-1**.** The *a*-QD-TA in S_0 state (Gaussian16).
Energy = -1029.819658 hartree

C	1.42234200	1.54404500	-0.00007000
C	0.68689800	2.74900300	-0.00005100
C	-0.68019000	2.70418400	0.00005900
C	-1.38325800	1.48562000	0.00012800
C	-0.65684200	0.28120500	0.00004700
C	0.77519000	0.29251600	-0.00003600
N	2.78743000	1.60298800	-0.00015400
C	3.60069400	0.49452000	-0.00008900
C	3.01674700	-0.78376300	0.00001900
C	1.55775500	-0.93748700	0.00003200
C	3.85167800	-1.91099000	0.00008800
C	5.22366600	-1.77402400	0.00005100
C	5.79352100	-0.49240600	-0.00005800
C	4.99695700	0.63356000	-0.00013000
N	-1.31510100	-0.90682400	-0.00003200
C	-2.68635300	-1.00657700	0.00000200
C	-3.47892800	0.15615000	0.00010400
C	-2.84895000	1.48558300	0.00032500
C	-4.87502300	0.02718300	0.00003600
C	-5.47103600	-1.21677700	-0.00009900
C	-4.67088200	-2.36897900	-0.00017700
C	-3.29490000	-2.27204600	-0.00013400
H	-1.26471100	3.61450200	0.00011800
H	1.21456400	3.69454600	-0.00008600
O	1.03872000	-2.06956100	0.00026500
O	-3.50934200	2.52780900	-0.00005800
H	3.21923600	2.51446400	-0.00010300
H	3.38205700	-2.88493000	0.00016500
H	5.86034000	-2.64855100	0.00010200
H	6.87029900	-0.38128700	-0.00008700
H	5.43846900	1.62275900	-0.00021400
H	-0.71359000	-1.73407300	-0.00001000
H	-5.46252300	0.93537300	0.00011800
H	-6.54926500	-1.30709700	-0.00013700
H	-5.13473100	-3.34728600	-0.00028600
H	-2.67598800	-3.16067200	-0.00021900

Corresponding Frequencies (in cm^{-1}):
36.25 48.06 89.05 101.46 118.37 137.22 154.62 223.94 240.62 251.63 270.90 280.10 302.35 331.72 371.82 420.13 421.33 422.96 454.94 458.50 494.27 512.88 517.10 526.98 545.12 567.60 586.79 611.96 614.90 631.48 651.22 658.19 663.30 671.64 708.17 726.07 743.40 772.93 774.15 784.87 795.66 822.00 844.19 856.55 864.03 889.83 900.63 944.34 967.54 968.65 986.71 988.67 989.64 1033.96 1058.10 1064.69 1104.29 1138.36 1148.31 1185.34 1186.20 1194.26 1210.03 1232.86 1245.14 1281.15 1287.99 1296.45 1320.16 1331.52 1339.84 1370.28 1375.75 1391.33 1426.25 1462.22 1479.15 1496.34 1517.07 1519.12 1544.82 1573.13 1603.92 1621.82 1627.45

1636.04 1643.47 1656.51 1664.17 1678.73 3175.56 3177.05 3177.52 3187.86 3189.76 3196.90 3200.98 3206.78 3208.50 3213.96 3346.11 3607.09

S6-2. The *a*-QD-TA in S_1 state (Gaussian16).
Energy = -1029.711760 hartree

C	-1.41424300	1.59492900	0.00000700
C	-0.67131400	2.79867100	0.00001500
C	0.70974400	2.79128700	-0.00002900
C	1.39393000	1.56021700	-0.00006900
C	0.64883100	0.36702100	-0.00005500
C	-0.77123700	0.34960500	-0.00003400
N	-2.78648800	1.62418100	0.00004000
C	-3.58066700	0.48313000	0.00003700
C	-2.95097000	-0.79050300	-0.00001600
C	-1.51599800	-0.89239800	-0.00005800
C	-3.77345500	-1.93340800	-0.00002700
C	-5.15788700	-1.81744800	0.00001500
C	-5.75847500	-0.56173500	0.00006900
C	-4.96645900	0.59106900	0.00007900
N	1.27012900	-0.85321700	-0.00003700
C	2.60625100	-1.00532300	-0.00001600
C	3.45083500	0.14105300	-0.00004200
C	2.85958600	1.49140900	-0.00014900
C	4.82873800	-0.04448900	0.00002200
C	5.37268400	-1.32394900	0.00009400
C	4.53652900	-2.45200100	0.00010800
C	3.16872700	-2.30463900	0.00005600
H	1.27919600	3.70830500	-0.00003900
H	-1.20612900	3.74125400	0.00004400
O	-0.91506700	-2.03859400	-0.00012900
O	3.55697800	2.50830900	0.00004400
H	-3.24837100	2.51979700	0.00007800
H	-3.29067500	-2.90006900	-0.00006900
H	-5.77188300	-2.70929300	0.00000600
H	-6.83647700	-0.46915800	0.00010200
H	-5.42670500	1.57220600	0.00011900
H	0.49984800	-1.64657800	0.00000500
H	5.46405600	0.83079100	0.00000200
H	6.44675200	-1.45177400	0.00013600
H	4.96988200	-3.44310700	0.00016600
H	2.50881000	-3.16183500	0.00007500

Corresponding Frequencies (in cm^{-1}):
37.14 53.28 90.29 102.21 103.92 136.17 149.76 181.87 240.68 247.32 251.47 252.21 277.34 321.99 356.87 367.25 390.83 401.86 412.81 436.59 449.53 456.91 483.21 498.33 517.84 522.61 543.91 550.99 569.34 593.83 618.60 621.89 643.77 650.07 663.87 675.79 718.80 736.06 764.70 776.90 781.75 809.94 824.03 856.99 871.48 872.09 879.95 885.83 927.47 949.87 978.28 986.50 1000.83 1048.92 1055.13 1084.59 1113.50 1124.85 1141.39 1146.56 1175.20 1182.80 1190.90 1208.43 1213.02 1236.01 1253.00 1260.30 1282.57 1303.81 1328.77 1344.03 1360.85 1372.73 1396.00 1429.57 1450.73 1469.33 1486.52 1494.85 1501.67 1511.95 1521.55 1562.78 1574.42

1594.18 1599.35 1619.31 1631.36 1650.82 2258.07 3167.32 3174.64 3178.15 3193.02 3198.10 3201.43 3209.23 3215.06 3215.27 3229.59 3619.25

S6-3. The *a*-QD-TB in S_1 state (Gaussian16).
Energy = -1029.721939 hartree

C	1.39371900	1.55846000	-0.00001500
C	0.65089100	2.77970100	-0.00000900
C	-0.70908200	2.75442600	-0.00004000
C	-1.39678900	1.50852900	-0.00006400
C	-0.68032500	0.29844500	-0.00007100
C	0.75727800	0.32083100	-0.00010000
N	2.76402000	1.62002900	0.00005300
C	3.58278500	0.50649900	0.00006900
C	2.97045600	-0.77963800	-0.00002500
C	1.56215600	-0.87543000	-0.00013200
C	3.81598100	-1.91644800	-0.00004200
C	5.19112800	-1.77216600	0.00005100
C	5.77324000	-0.50211500	0.00015500
C	4.96770300	0.63571600	0.00016000
N	-1.28736300	-0.92094100	-0.00000600
C	-2.62810800	-0.99910900	0.00003300
C	-3.47112300	0.15385400	0.00000900
C	-2.86073200	1.48214500	-0.00004900
C	-4.85688500	-0.00544100	0.00005500
C	-5.42408200	-1.26979800	0.00011900
C	-4.60258300	-2.41149600	0.00014000
C	-3.23447800	-2.28559000	0.00009900
H	-1.29471500	3.66211300	-0.00003100
H	1.19013000	3.71930900	0.00003400
O	1.01548100	-2.09826100	-0.00033500
O	-3.52608000	2.52737800	-0.00001700
H	3.19960400	2.52934700	0.00012900
H	3.35900900	-2.89492900	-0.00012300
H	5.82194300	-2.65185100	0.00004200
H	6.84964200	-0.39528700	0.00022500
H	5.41205700	1.62379300	0.00023100
H	0.01932100	-1.96042900	-0.00057800
H	-5.47417200	0.88308400	0.00004100
H	-6.50041100	-1.38059800	0.00015300
H	-5.05280700	-3.39587000	0.00019000
H	-2.58831600	-3.15346800	0.00011400

Corresponding Frequencies (in cm^{-1}):
36.30 50.30 83.63 104.47 119.45 137.25 145.05 186.70 234.00 251.95 258.66 274.46 276.31 326.41 358.07 361.50 402.98 417.77 423.37 459.23 463.04 469.09 493.22 507.23 525.28 536.47 542.08 561.37 568.38 606.95 615.04 647.60 650.33 660.46 662.93 708.48 733.44 737.96 765.14 776.94 777.37 826.00 832.91 868.70 873.53 888.31 897.74 924.40 933.20 935.20 959.02 977.54 983.62 1031.00 1049.77 1059.96 1088.92 1125.26 1136.19 1153.60 1170.68 1185.07 1195.14 1211.45 1227.87 1243.63 1273.13 1280.93 1302.12 1326.49 1350.41 1353.53 1370.26 1387.20 1404.16 1438.91 1455.83 1477.59 1482.62 1488.10 1503.28 1526.48 1531.68 1561.35 1589.14

1592.81 1607.60 1610.63 1632.52 1654.70 2999.57 3174.08 3175.84 3183.56 3183.73 3194.44 3202.64 3202.69 3208.47 3222.09 3222.89 3612.54

S6-4. The TS state for the *a*-QD on the S_1 state (Gaussian16).
Energy = -1029.711751 hartree

C	1.41403900	1.59990800	0.00002800
C	0.67016800	2.80465300	0.00019500
C	-0.70946000	2.79544300	0.00014200
C	-1.39448600	1.56300100	0.00014700
C	-0.65008600	0.37095000	0.00011800
C	0.76982400	0.35721800	0.00032500
N	2.78652700	1.62615700	-0.00010900
C	3.57743100	0.48270200	-0.00009200
C	2.94435000	-0.79043000	-0.00007600
C	1.51234600	-0.88480100	-0.00003100
C	3.76452900	-1.93612900	-0.00003400
C	5.14861600	-1.82307500	-0.00008100
C	5.75233100	-0.56849500	-0.00013000
C	4.96336700	0.58632800	-0.00014000
N	-1.26221500	-0.85377300	0.00018200
C	-2.59713000	-1.00551400	0.00005100
C	-3.44615900	0.13911000	-0.00003800
C	-2.85941800	1.49140500	0.00002400
C	-4.82364600	-0.04975300	-0.00014800
C	-5.36471900	-1.33033600	-0.00017900
C	-4.52585500	-2.45664300	-0.00008900
C	-3.15872000	-2.30625100	0.00002500
H	-1.27981100	3.71202500	0.00024100
H	1.20438100	3.74755800	0.00023100
O	0.90200300	-2.03133000	-0.00000800
O	-3.55966400	2.50677400	-0.00002100
H	3.25105600	2.52041800	-0.00027500
H	3.28000700	-2.90190700	0.00001100
H	5.76067500	-2.71624000	-0.00006500
H	6.83054100	-0.47868400	-0.00016300
H	5.42631500	1.56619100	-0.00015300
H	-0.45248400	-1.64841900	0.00029000
H	-5.46108200	0.82403800	-0.00020800
H	-6.43852000	-1.46063500	-0.00027100
H	-4.95729300	-3.44863700	-0.00011100
H	-2.49659700	-3.16169000	0.00009500

Corresponding Frequencies (in cm^{-1}):
-241.56 37.59 54.13 90.66 104.53 136.10 150.17 152.88 180.74 240.71 250.81 251.04 277.58 298.66 333.43 367.71 391.67 396.49 413.67 435.18 437.18 458.09 481.15 498.56 509.23 518.64 545.53 548.06 566.68 583.38 616.42 624.11 638.51 650.69 660.71 668.12 720.58 735.93 765.37 777.63 779.08 784.07 824.16 851.03 869.48 872.11 879.17 889.57 925.21 950.30 979.16 982.01 986.67 1046.94 1054.29 1084.79 1093.27 1122.36 1138.04 1168.18 1181.45 1186.12 1188.17 1195.29 1213.77 1235.25 1251.69 1260.48 1283.00 1303.83 1329.29 1345.35 1359.21 1374.91 1397.12 1428.84 1443.87 1470.48 1486.16 1494.07 1499.87 1508.42 1521.82 1559.99 1572.50

1592.24 1599.37 1620.93 1623.44 1650.75 2084.53 3167.44 3175.07 3178.35 3192.67 3198.38 3201.47 3209.18 3214.75 3215.39 3228.64 3619.52

S6-5. The *a*-QD-TB in S_1 state (ORCA).
Energy = -1029.727274827880 hartree

C	1.39499218285006	1.55906263730267	-0.00004945997760
C	0.65194403963936	2.77799810496101	-0.00003398213220
C	-0.70990947829672	2.75434132001168	-0.00012087837884
C	-1.39756427777613	1.51050608423367	-0.00016680759943
C	-0.68044937220096	0.29929787192584	-0.00017892316422
C	0.75761283495211	0.31974794406829	-0.00019658468474
N	2.76535831486421	1.62089945308780	0.00010611405362
C	3.58255795348256	0.50692788386263	0.00013351244646
C	2.97064500398037	-0.77961533864917	-0.00006915930315
C	1.56148880876079	-0.87471892447319	-0.00028647335836
C	3.81577163673145	-1.91631447764591	-0.00009973818422
C	5.19182321576996	-1.77250694364240	0.00009238694100
C	5.77380962092970	-0.50233228686783	0.00030909662787
C	4.96806671861028	0.63573173475504	0.00032119057660
N	-1.28853047173572	-0.91871844316508	-0.00002582286365
C	-2.62959386505362	-0.99734632057551	0.00005824179467
C	-3.47289703612691	0.15528822470120	-0.00000682048172
C	-2.86173090733984	1.48300098075477	-0.00016572666074
C	-4.85867789531255	-0.00536288296247	0.00010196004869
C	-5.42454813932676	-1.27097015568874	0.00025365637696
C	-4.60245028096767	-2.41200793001555	0.00030894879445
C	-3.23390241737941	-2.28452584102993	0.00021446325342
H	-1.29195585025697	3.66434295306479	-0.00010346144337
H	1.19211510257672	3.71677294182170	0.00005138219165
O	1.01582182225156	-2.10002050498829	-0.00071856368615
O	-3.52735826453287	2.52870996133348	-0.00006431299346
H	3.20236576159740	2.53039714528879	0.00023560307202
H	3.36015669928775	-2.89554346660965	-0.00026942034478
H	5.82223448916183	-2.65251625780721	0.00007290485936
H	6.85030226111759	-0.39553617777204	0.00045490647356
H	5.41000229026165	1.62484113567091	0.00047094498558
H	0.01997963191286	-1.96502804354227	-0.00123747042734
H	-5.47972510680361	0.88062457685018	0.00006060236488
H	-6.50083003110001	-1.38234825412273	0.00033155309417
H	-5.05189675701912	-3.39672815550800	0.00042724368234
H	-2.58653523750934	-3.15155654862847	0.00025389404669

Corresponding Frequencies (in cm^{-1}):
34.19 45.11 81.03 105.83 118.95 134.56 142.71 189.23 233.43 252.09 260.58 275.92 276.46 324.71 356.03 361.75 402.46 419.35 421.08 459.03 463.23 469.94 493.56 504.55 524.09 536.37 540.78 562.80 566.22 609.18 614.64 647.48 648.93 656.32 663.08 708.54 733.69 738.36 760.79 776.07 776.17 826.35 833.29 866.00 874.86 892.50 897.99 912.34 924.87 935.56 959.62 973.08 984.40 1030.37 1048.18 1060.16 1088.42 1124.60 1134.51 1153.90 1172.68 1183.12 1192.95 1214.00 1228.96 1244.01 1276.36 1281.10 1302.55 1326.40 1349.13 1353.14 1368.68 1386.51 1403.15 1438.27 1450.88 1478.36 1481.72 1487.66 1501.06 1522.07 1534.43 1560.66 1589.44

1590.91 1603.25 1609.04 1628.85 1650.25 3005.62 3173.25 3182.08 3182.56 3183.07 3193.51 3200.45 3201.37 3206.19 3218.84 3220.96 3605.48

S6-6. The *a*-QD-TB in T_1 state (ORCA).
Energy = -1029.741764537632 hartree

C	1.40087723016653	1.57185339254008	-0.00001768168273
C	0.66116240524384	2.76856212960150	-0.00002235492296
C	-0.71957315403851	2.75000547705269	-0.00003608060312
C	-1.40241024048334	1.52749759755416	-0.00001809309468
C	-0.68032119175123	0.31023903919035	-0.00002045393981
C	0.75787372198252	0.31744311233389	-0.00006585472767
N	2.77325824010293	1.62366290673254	0.00003479937126
C	3.59105663517233	0.50567735964487	0.00005500560026
C	2.97671178092952	-0.77384691172648	-0.00001044803781
C	1.56030734960157	-0.86163067206872	-0.00009421687218
C	3.81259832918098	-1.90988331012282	-0.00002399328256
C	5.19139514431269	-1.77534448663776	0.00004667534752
C	5.77724534817706	-0.51003914860373	0.00012525117061
C	4.97482022145222	0.63004047796859	0.00012631510153
N	-1.29563432523942	-0.90757562249343	0.00004788162487
C	-2.63864563872145	-0.99225174051950	0.00006635590803
C	-3.48340353361774	0.15701630173078	0.00004467405269
C	-2.87209836881025	1.49457346215335	0.00007484993181
C	-4.86525835028185	-0.00771907784976	0.00003430838367
C	-5.42567495637225	-1.28000571782755	0.00006058101916
C	-4.60159279455788	-2.41764694495468	0.00009273553316
C	-3.23144321318787	-2.28247617003715	0.00009413408158
H	-1.28806986192227	3.66820356539266	-0.00002431969126
H	1.19588608414730	3.71105706246153	-0.00000322021144
O	1.01976898731277	-2.09791034019719	-0.00027570567246
O	-3.54990094908675	2.52590385315557	-0.00006645911921
H	3.21364262878568	2.53067727450336	0.00007475453598
H	3.35257967484266	-2.88644919350334	-0.00008473077503
H	5.81572498674468	-2.65906545232040	0.00004103965269
H	6.85346566664266	-0.40631992828521	0.00017848712319
H	5.42055466445120	1.61688676470078	0.00017747006881
H	0.03268842420514	-1.97647832388156	-0.00046011677350
H	-5.49198735166227	0.87472834008314	0.00002416801627
H	-6.50217262898769	-1.39483060344719	0.00006290235048
H	-5.04718828816138	-3.40432555502724	0.00011415115340
H	-2.57774967657210	-3.14543491729611	0.00011218937947

Corresponding Frequencies (in cm^{-1}):
35.90 48.34 84.93 100.90 119.66 136.76 144.56 205.04 244.93 251.72 270.13 275.56 281.64 325.56 351.10 359.26 395.79 421.27 422.16 457.05 473.55 486.40 490.05 501.70 520.45 529.51 534.09 559.67 563.63 609.52 614.34 621.25 639.44 648.21 665.44 705.23 737.44 741.47 768.69 776.41 797.89 829.33 830.07 832.42 875.21 875.58 899.64 901.46 918.17 936.29 957.53 980.01 986.17 1024.28 1047.41 1058.15 1080.06 1124.74 1129.82 1166.71 1170.05 1180.45 1193.49 1209.01 1225.38 1244.51 1276.04 1296.21 1300.58 1319.09 1341.40 1346.83 1359.66 1365.11 1381.21 1414.35 1436.68 1472.20 1474.41 1485.86 1508.79 1521.72 1534.10 1561.01 1570.70

1591.33 1598.88 1614.62 1617.82 1642.98 3160.93 3175.99 3177.78 3180.76 3187.05 3190.13 3198.30 3202.86 3205.21 3221.02 3223.78 3612.74

S6-7. The *a*-QD-TB in T_2 state (ORCA).
Energy = -1029.717542924511 hartree

C	1.42505817672298	1.59816191666357	-0.00000836051057
C	0.67718192602705	2.78859930425001	-0.00000948929175
C	-0.69793744604861	2.74832802236833	-0.00005961408636
C	-1.41106057021657	1.52271124447234	-0.00008368820932
C	-0.66646147944836	0.30847762299371	-0.00006241569134
C	0.75424113791452	0.33458564825946	-0.00008611279438
N	2.78716509138857	1.62669830156468	0.00006073717101
C	3.57521033360381	0.51517361596407	0.00007627479349
C	2.95844230833864	-0.76914603512559	-0.00001649346240
C	1.54295588492844	-0.85226392421604	-0.00013820046382
C	3.78639853385311	-1.91717372601989	-0.00003216125879
C	5.15318301476986	-1.79432124532013	0.00005983883666
C	5.75061903827889	-0.51315843136937	0.00015864565227
C	4.97701117850827	0.62421201302534	0.00016387333749
N	-1.27436298448722	-0.90768160517194	0.00003442146273
C	-2.62878378161272	-1.00393521235499	0.00006036155186
C	-3.46628782921743	0.15086795314160	-0.00000777735842
C	-2.85042349607587	1.48202446580706	-0.00009733988840
C	-4.84481977864077	-0.02460787347910	0.00001965208700
C	-5.40544958681306	-1.31297017797925	0.00011252590094
C	-4.59150467732645	-2.43951646729827	0.00018788273987
C	-3.20693886965367	-2.29147942685179	0.00016729256091
H	-1.27271036873423	3.66361579881957	-0.00006272400717
H	1.19797368134825	3.73898353777444	0.00003057818323
O	0.97580395401622	-2.03851049588516	-0.00038303863298
O	-3.54609259954063	2.53610321802955	-0.00007530593668
H	3.24029472364838	2.53092035479064	0.00011604221537
H	3.31607291266147	-2.89009642863095	-0.00011347717325
H	5.77723241985887	-2.67764346836948	0.00005199758373
H	6.82884584187075	-0.42373763714868	0.00022499554409
H	5.43343282897830	1.60648228532353	0.00023013077616
H	-0.04896051343232	-1.87263623828221	-0.00064023589145
H	-5.47605492114368	0.85330702803884	-0.00002904832108
H	-6.48266052594620	-1.42529896602996	0.00012703931043
H	-5.02912968513511	-3.42924829738490	0.00026410348064
H	-2.54899087324346	-3.15103267436906	0.00022408979031

Corresponding Frequencies (in cm^{-1}):
37.29 55.22 88.06 107.48 122.15 135.50 152.85 212.58 244.68 251.99 280.84 291.43 297.66 314.94 358.25 385.59 404.04 422.22 430.96 461.34 486.23 492.52 504.82 526.97 539.98 564.09 565.52 607.97 611.28 614.73 632.09 646.58 648.72 661.90 678.33 703.95 746.19 756.67 762.41 772.94 804.81 828.77 840.39 843.75 888.72 904.46 917.59 930.90 941.29 954.80 957.62 979.44 1023.35 1043.07 1047.62 1061.43 1099.15 1110.92 1144.49 1163.60 1167.40 1173.33 1184.55 1218.77 1253.21 1268.81 1271.64 1286.36 1309.22 1331.82 1352.24 1365.10 1377.49 1390.28 1424.40 1443.05 1448.89 1463.74 1471.61 1487.23 1502.60 1509.91 1528.82 1536.34 1551.97

1565.91 1585.19 1612.71 1628.40 1858.82 2432.12 3174.72 3176.06 3179.06 3186.42 3192.72 3198.50 3203.81 3205.40 3214.56 3217.47 3578.38

S6-8. The *a*-QD-TB in T_3 state (ORCA)
Energy = -1029.707559853226 hartree

C	1.40065638480736	1.58382192619701	0.00000279772992
C	0.67100677622835	2.78570377432840	0.00000688851824
C	-0.70546942230417	2.74026709851407	-0.00002654967748
C	-1.40180385848434	1.51575549569218	-0.00006571406572
C	-0.67228798647456	0.29521369574057	-0.00005829615156
C	0.75133810386792	0.33058050769355	-0.00005813860426
N	2.77672689553480	1.60921849065904	0.00004642629162
C	3.57209628674849	0.50012376521266	0.00004329697435
C	2.95917312020554	-0.78537716786671	-0.00001380210454
C	1.54231608567099	-0.86930911263630	-0.00006675679933
C	3.79872777160067	-1.91627452770539	-0.00003267812594
C	5.18878745611550	-1.77266023497213	0.00002501916371
C	5.77472899796132	-0.50920930565525	0.00009632240337
C	4.96929912201229	0.63023121633324	0.00011310143316
N	-1.26302988089340	-0.92556122076918	-0.00006405218185
C	-2.61522831142159	-1.01137628575436	-0.00002599888717
C	-3.45358005911213	0.14952390216267	-0.00001968917577
C	-2.85977980751907	1.48048148154081	-0.00007501683078
C	-4.84611059190186	-0.01806822276109	0.00004331967078
C	-5.41113682644575	-1.28195584674380	0.00010299469626
C	-4.58989391283120	-2.42314304216916	0.00009156134338
C	-3.21673743875232	-2.29258196648663	0.00002395773072
H	-1.28780472695717	3.65104379170597	-0.00002768953376
H	1.19780017399869	3.73182149232120	0.00004202911578
O	0.96449919310241	-2.05299381083833	-0.00013890269058
O	-3.54406837823293	2.52757763247337	-0.00003630543731
H	3.22700989724420	2.51614527727122	0.00008813684212
H	3.34644918042151	-2.89731687103608	-0.00008160220282
H	5.81311219012990	-2.65661912934958	0.00001083883586
H	6.85052265648787	-0.40409713187407	0.00014495634322
H	5.40961342883657	1.61986590079975	0.00016739754067
H	-0.07787949147580	-1.86151419787974	-0.00015230176399
H	-5.46445713578568	0.86931698790073	0.00004665500867
H	-6.48807238975206	-1.39374101854802	0.00015595081439
H	-5.03740666185612	-3.40893561305860	0.00013900280216
H	-2.57062384077422	-3.16116373044200	0.00001784097448

Corresponding Frequencies (in cm^{-1}):
38.10 51.97 91.12 103.83 123.23 134.87 151.84 191.12 235.32 251.38 252.87 279.14 290.57 319.34 351.70 360.72 401.01 419.30 426.80 459.42 467.41 490.05 498.62 510.24 539.97 556.32 569.29 574.02 601.20 612.35 614.91 633.74 642.70 649.39 660.12 717.12 723.42 730.91 758.00 774.63 775.44 815.08 833.67 849.72 885.34 898.65 906.26 936.80 941.72 944.95 949.39 964.66 1024.77 1045.85 1064.85 1089.05 1089.71 1120.42 1129.00 1158.04 1178.03 1189.91 1199.14 1205.00 1240.89 1256.43 1275.27 1285.57 1301.56 1323.71 1357.38 1362.84 1390.29 1401.58 1410.02 1449.80 1461.72 1464.11 1483.03 1493.34 1504.12 1517.00 1525.40 1537.07 1553.65

1583.65 1599.15 1605.47 1629.35 2020.90 2133.68 3175.36 3181.49 3182.25 3185.05 3191.74 3195.66 3203.76 3210.66 3211.78 3220.37 3567.35

S6-9. The TQB-TA in S_0 state (Gaussian16).
Energy = -1428.575206 hartree

C	0.29102400	1.37666900	0.00476500
C	1.36897000	0.43035300	0.01723500
C	1.04689000	-0.94036100	0.00465500
C	-0.31163300	-1.40075400	0.01710300
C	-1.33762900	-0.43643100	0.00466400
C	-1.05708700	0.97022600	0.01718400
N	0.59920200	2.69219000	0.00205700
C	-0.33901400	3.69388000	-0.00038200
C	-1.70411600	3.36384100	-0.00144900
C	-2.12006900	1.96071400	0.00375500
C	-2.65460900	4.39583500	-0.00448500
C	-2.25698100	5.71609500	-0.00722500
C	-0.88980800	6.03323600	-0.00676200
C	0.06470700	5.03804600	-0.00363600
N	-2.63102600	-0.82713700	0.00192900
C	-3.02960000	-2.14044900	-0.00044100
C	-2.06130900	-3.15774500	-0.00152100
C	-0.63811300	-2.81653600	0.00363100
C	-2.48009000	-4.49682900	-0.00448500
C	-3.82234200	-4.81237300	-0.00714400
C	-4.78044400	-3.78682500	-0.00667200
C	-4.39562000	-2.46268100	-0.00362000
N	2.03207800	-1.86500600	0.00185100
C	3.36869800	-1.55333500	-0.00050000
C	3.76548100	-0.20609800	-0.00141800
C	2.75825100	0.85566100	0.00388400
C	5.13453200	0.10093600	-0.00439600
C	6.07900800	-0.90364400	-0.00720700
C	5.67000700	-2.24618700	-0.00688200
C	4.33087400	-2.57510600	-0.00383200
O	-3.33426200	1.65954000	0.00023700
O	0.22973100	-3.71755100	0.00011700
O	3.10439800	2.05779000	0.00033700
H	1.61189800	2.88577700	0.00152900
H	-3.70036200	4.12126900	-0.00463400
H	-2.99427100	6.50789200	-0.00948200
H	-0.57779400	7.06981100	-0.00897700
H	1.12039500	5.27774600	-0.00341800
H	-3.30478900	-0.04672200	0.00137600
H	-1.71976400	-5.26551200	-0.00462400
H	-4.13953800	-5.84674200	-0.00934000
H	-5.83419200	-4.03473300	-0.00881800
H	-5.13092600	-1.66815600	-0.00337300
H	1.69334100	-2.83881300	0.00113400
H	5.41997500	1.14379400	-0.00441700
H	7.13338300	-0.66109200	-0.00940800

H	6.41163000	-3.03475500	-0.00914900
H	4.01043900	-3.60914900	-0.00370400

Corresponding Frequencies (in cm^{-1}):

36.72 40.66 41.16 85.44 95.95 96.62 121.26 121.56 131.81 152.69 153.06 214.15 247.00
247.63 258.84 273.44 281.06 281.13 304.00 318.11 318.67 379.08 379.61 410.17 425.45 426.73
427.18 506.29 506.44 507.11 518.38 518.48 530.74 558.03 558.07 597.50 609.39 609.65 632.08
632.21 650.54 656.71 656.85 660.23 660.29 662.86 709.48 710.11 731.39 731.66 772.56 774.46
775.20 780.79 780.89 806.32 847.27 862.62 863.53 865.60 887.50 890.17 892.55 893.44 901.47
901.56 971.78 971.89 972.21 975.41 987.21 987.26 987.64 1040.46 1040.54 1055.41 1068.55
1068.89 1139.31 1139.44 1145.10 1184.80 1185.54 1185.78 1203.91 1204.05 1226.64 1252.78
1252.87 1275.94 1285.05 1285.28 1289.36 1341.30 1342.58 1342.63 1350.73 1376.39 1378.54
1378.60 1386.06 1386.41 1462.91 1474.55 1474.64 1482.62 1511.73 1511.99 1521.67 1542.40
1542.59 1589.37 1596.90 1596.92 1601.60 1631.61 1631.71 1647.78 1648.27 1653.18 1659.85
1666.88 1667.08 3181.64 3181.75 3181.95 3191.68 3191.84 3192.00 3195.86 3200.60 3200.69
3201.13 3205.11 3205.90 3212.57 3213.56 3213.91

S6-10. The TQB-TA in S_1 state (Gaussian16).

Energy = -1428.452267 hartree

C	0.95254000	-1.03782700	-0.00193500
C	-0.42685200	-1.37038700	0.01222700
C	-1.37405800	-0.30649200	-0.00209700
C	-0.97291500	1.05490800	0.01204000
C	0.42184300	1.34292000	-0.00206500
C	1.40011400	0.31483200	0.01210500
N	1.87135400	-2.04344800	-0.00090800
C	3.22207700	-1.85252400	-0.00056600
C	3.73680500	-0.53428100	-0.00133200
C	2.82867100	0.60603400	0.00212800
C	5.12550600	-0.35479100	-0.00134800
C	5.98043000	-1.44483600	-0.00193700
C	5.45871200	-2.74645000	-0.00197300
C	4.09199000	-2.95514800	-0.00165100
N	0.83385200	2.64105400	-0.00119900
C	-0.00649700	3.71586300	-0.00070000
C	-1.40569300	3.50252400	-0.00141700
C	-1.93910100	2.14621100	0.00192800
C	-2.25508300	4.61567200	-0.00137900
C	-1.73788600	5.90096400	-0.00199100
C	-0.34981800	6.09989600	-0.00209200
C	0.51400600	5.02020700	-0.00182400
N	-2.70451800	-0.59825700	-0.00122000
C	-3.21533800	-1.86283300	-0.00070700
C	-2.33146200	-2.96826700	-0.00134300
C	-0.88986300	-2.75284100	0.00219300
C	-2.87084700	-4.26036800	-0.00127300
C	-4.24245500	-4.45498600	-0.00195700
C	-5.10857200	-3.35196500	-0.00214900
C	-4.60546000	-2.06431200	-0.00190600
O	3.27398800	1.78164500	-0.00015600
O	-3.18037300	1.94376900	-0.00036100
O	-0.09515300	-3.72674800	0.00002600

H	1.44703000	-2.98449900	-0.00083700
H	5.50689400	0.65671400	-0.00104400
H	7.05165500	-1.29330900	-0.00211200
H	6.12811600	-3.59675800	-0.00251400
H	3.68038300	-3.95643900	-0.00181000
H	1.86128300	2.74371500	-0.00113300
H	-3.32181200	4.44032000	-0.00105600
H	-2.40459900	6.75304100	-0.00213600
H	0.05211800	7.10463400	-0.00267200
H	1.58699700	5.16404900	-0.00206000
H	-3.30622800	0.24146000	-0.00131500
H	-2.18552300	-5.09653200	-0.00088300
H	-4.64731200	-5.45826500	-0.00208800
H	-6.17968700	-3.50614000	-0.00278300
H	-5.26659200	-1.20710000	-0.00220900

Corresponding Frequencies (in cm^{-1}):
34.98 39.20 40.96 77.43 80.60 81.33 92.32 93.47 125.57 146.77 149.35 211.11 245.63 246.08 257.71 264.79 266.05 274.17 287.91 308.80 309.38 366.97 368.61 406.75 411.16 412.12 412.58 489.21 490.01 492.63 493.10 499.54 512.91 535.94 537.08 540.26 540.77 588.39 609.98 610.34 613.61 615.13 615.43 644.90 645.09 656.66 659.03 689.97 690.55 704.05 752.89 753.53 754.93 755.97 756.30 803.81 835.89 848.17 849.60 850.40 876.77 877.59 884.01 896.23 897.43 899.81 947.78 947.88 948.12 967.02 967.68 968.80 972.00 1014.28 1014.96 1053.77 1055.87 1056.76 1134.04 1134.63 1139.38 1139.99 1142.80 1183.19 1183.44 1184.34 1200.25 1200.96 1222.56 1260.75 1261.57 1288.68 1292.87 1293.67 1299.86 1326.65 1327.13 1334.12 1364.00 1364.37 1365.46 1372.88 1410.16 1410.81 1431.51 1455.73 1458.44 1474.85 1507.46 1508.08 1508.85 1519.05 1519.58 1539.59 1563.78 1580.24 1580.52 1606.45 1621.34 1621.87 1625.27 1625.83 1628.00 3123.13 3133.41 3177.70 3183.11 3183.20 3183.32 3192.04 3192.19 3192.26 3202.85 3202.90 3203.16 3214.73 3215.70 3216.33

S6-11. The TQB-TA in S_1 state (ORCA).
Energy = -1428.458570584337 hartree

C	0.95261288093839	-1.03569127262845	-0.00224878305149
C	-0.42724415178015	-1.36923818341811	0.01142932190340
C	-1.37508464235693	-0.30544789108017	-0.00309223346782
C	-0.97322377861562	1.05589015994261	0.01062734637635
C	0.42239208806900	1.34500967485886	-0.00269782464299
C	1.40055849304759	0.31660284403347	0.01132804219791
N	1.87078232388664	-2.04142365641220	-0.00059544544759
C	3.22125115868204	-1.85224896514278	0.00016502346682
C	3.73705130145617	-0.53423884104063	-0.00108786096950
C	2.82924508083481	0.60587196390822	0.00227883025463
C	5.12633455995023	-0.35687773852944	-0.00126129939712
C	5.97986712692704	-1.44851786803357	-0.00131013800798
C	5.45682986949728	-2.75002235259079	-0.00049714805990
C	4.08965165557080	-2.95654050630274	-0.00001642854985
N	0.83305619146770	2.64357257231608	-0.00089421752690
C	-0.00721639356028	3.71769389504168	-0.00032643441758
C	-1.40630829156843	3.50474052861488	-0.00269775142319
C	-1.93897460449599	2.14810685697304	0.00007252253217
C	-2.25558632675739	4.61852677575420	-0.00309903623255
C	-1.73829519728465	5.90387943406614	-0.00226019710294

C -0.34966813182595 6.10226485302872 -0.00027519832753
C 0.51372568705415 5.02229454593791 0.00042887068941
N -2.70505314087741 -0.59928557973289 -0.00187695443746
C -3.21465210525275 -1.86470311664139 -0.00052131556308
C -2.32985387484964 -2.96927820265426 -0.00124739285367
C -0.88898709996784 -2.75149751297792 0.00220357254923
C -2.86877385094999 -4.26210424193875 -0.00068225394396
C -4.24055521776781 -4.45770484040900 -0.00058724270676
C -5.10743852947677 -3.35511843299383 -0.00034618976724
C -4.60463302562188 -2.06690184608737 -0.00058242470111
O 3.27613332798732 1.78172632205988 0.00116554125406
O -3.18042116804470 1.94630047863415 -0.00285757298780
O -0.09262442813859 -3.72585455445014 0.00145340215463
H 1.44835620491791 -2.98349927804927 -0.00079281101713
H 5.51178094987131 0.65321344601295 -0.00144592672046
H 7.05127139100809 -1.29794929037689 -0.00185800168489
H 6.12536669131079 -3.60102754008251 -0.00034191767453
H 3.67460036996885 -3.95642030282374 0.00046634464927
H 1.85900543433308 2.75149517494698 -0.00071511190970
H -3.32287208405424 4.44612428051551 -0.00411757379022
H -2.40499022635352 6.75597631413783 -0.00291319593132
H 0.05243915821458 7.10701562890683 0.00064247608380
H 1.58702255747687 5.16408795840555 0.00184185855922
H -3.31142768436043 0.23551778226263 -0.00302719139367
H -2.18498993377928 -5.09950535366494 -0.00042302036989
H -4.64445293175937 -5.46142007206523 -0.00053770969790
H -6.17854173007267 -3.50986962309983 -0.00004801383704
H -5.26476595289834 -1.20882442713130 -0.00052133505815

Corresponding Frequencies (in cm^{-1}):
21.89 33.42 34.36 76.59 77.53 79.96 87.25 88.01 123.95 147.06 148.18 210.44 244.24 244.66 256.80 263.94 265.05 273.11 286.45 309.79 310.28 365.37 365.80 404.88 411.42 412.96 413.56 488.41 489.50 490.92 491.42 499.08 510.77 533.66 535.44 540.05 540.94 588.29 609.54 610.74 613.35 615.22 616.18 643.89 644.31 655.33 659.60 691.05 692.59 703.63 749.22 750.59 750.73 751.90 752.99 793.95 835.79 844.63 845.59 848.58 874.24 876.33 880.38 883.06 883.30 884.36 949.47 949.56 949.95 965.85 966.15 966.46 971.67 1010.42 1010.93 1050.89 1052.90 1054.57 1129.82 1131.58 1134.79 1136.00 1138.23 1179.60 1180.29 1181.18 1195.04 1196.68 1221.17 1257.82 1258.91 1286.36 1289.32 1290.19 1297.64 1323.06 1324.23 1333.17 1362.38 1362.79 1363.75 1371.56 1406.91 1407.09 1430.14 1456.23 1458.94 1472.67 1505.32 1506.19 1507.10 1516.38 1516.96 1537.00 1560.17 1577.84 1578.02 1604.47 1616.73 1617.80 1621.82 1622.37 1623.54 3132.54 3146.55 3182.73 3182.90 3183.55 3187.85 3190.76 3191.38 3192.20 3201.08 3201.25 3201.63 3211.76 3211.89 3212.28

S6-12. The TQB-TA in T_2 state (ORCA)
Energy = -1428.473723064808 hartree
C -1.18622071748519 -0.76811124343387 0.00999248351757
C 0.08144066199754 -1.43697917160807 0.03138480977513
C 1.24646150601182 -0.64015023465723 0.01235798546304
C 1.20418547025695 0.78116400670357 0.02761109517185
C -0.05622192375439 1.39205278943361 0.00733174881467
C -1.27409867451395 0.63840000940265 0.01982758251031
N -2.31146608346639 -1.52074339920180 0.00696092883303

C	-3.57300465821751	-0.99353770182828	0.00100926830106
C	-3.74276718921578	0.40411820049138	-0.00185272595743
C	-2.58096528206396	1.28499287456933	0.00377911223585
C	-5.04534286993788	0.93174945689225	-0.00801540072244
C	-6.14055327584208	0.09668570674366	-0.01127004990432
C	-5.95788068878129	-1.29782246172190	-0.00883361157959
C	-4.69319637361341	-1.84290377446281	-0.00303270701493
N	-0.14285228898986	2.75480829217696	0.00567106560734
C	0.92933229164278	3.59479819369816	0.00291239323571
C	2.23701350320507	3.03603212702044	0.00094391902596
C	2.41211043132553	1.60690096465866	0.01118768129376
C	3.32718939881889	3.92483244536570	-0.00498511237879
C	3.12081082754244	5.29954839923061	-0.01101032466630
C	1.82679403198245	5.83519965763387	-0.00907050200307
C	0.72994946583644	4.98517506506300	-0.00326050470239
N	2.47125874255869	-1.25387999293148	0.01411464593532
C	2.65578365089048	-2.60259096940959	0.00682824691911
C	1.53266018462477	-3.45850231111736	0.00479387781264
C	0.18892481708278	-2.89137972227580	0.01758400863718
C	1.74233934061796	-4.84166958085752	-0.00638482874226
C	3.03749259153587	-5.36548349879341	-0.01742583309885
C	4.13865240739106	-4.51222873355349	-0.01491548471879
C	3.95992421267749	-3.13368122936302	-0.00323306802880
O	-2.72030521418785	2.52912662086717	-0.00195642693845
O	3.57627622870134	1.08934915980051	0.01106780637641
O	-0.82334594571473	-3.64331392209089	0.01971761110339
H	-2.13872823724639	-2.53679565474265	0.01064496718946
H	-5.15819334680719	2.00706377139666	-0.00965582952301
H	-7.14078088663609	0.50907791530482	-0.01557079031738
H	-6.81992133265061	-1.95262827611606	-0.01154424215720
H	-4.54588382982120	-2.91545804030140	-0.00136042090161
H	-1.11497085276180	3.10717817165382	0.00278124938725
H	4.32522899959995	3.51126521646443	-0.00583612175139
H	3.97434186298306	5.96586486830971	-0.01634220754093
H	1.68033202036923	6.90632061512754	-0.01346606028405
H	-0.28101745338854	5.37265725497974	-0.00238615278202
H	3.26037103485792	-0.58258186709965	0.01340255644892
H	0.87912666562011	-5.49184911978181	-0.00753357307251
H	3.18348736388686	-6.43767476082395	-0.02743391982530
H	5.14120645776109	-4.91930368920476	-0.02334618469222
H	4.80568995531753	-2.45827842761146	-0.00217296029092

Corresponding Frequencies (in cm^{-1}):

23.94 28.57 36.31 78.22 89.24 93.17 108.11 120.46 127.03 144.39 147.66 211.42 240.94
252.60 253.17 272.39 273.11 281.46 295.10 318.69 330.44 368.54 381.00 399.78 403.18 412.64
426.64 492.10 499.35 501.89 504.70 506.66 519.41 549.62 554.96 569.18 584.31 590.27 593.07
605.04 622.44 624.43 643.86 649.82 654.01 660.85 671.23 695.43 703.76 726.12 737.66 738.19
766.02 766.64 772.24 825.01 829.74 832.64 847.14 850.93 861.22 878.27 891.68 897.81 898.21
911.08 924.06 934.27 953.12 957.35 964.14 968.91 983.36 988.41 1019.69 1026.60 1050.64
1060.85 1064.70 1116.96 1127.63 1141.51 1169.30 1171.54 1182.71 1183.41 1199.47 1214.78
1219.08 1243.69 1264.61 1278.23 1284.59 1287.64 1312.80 1325.16 1337.58 1356.36 1361.32
1367.27 1372.42 1385.63 1410.03 1440.07 1465.25 1472.96 1474.71 1476.74 1492.70 1504.40

1509.05 1513.62 1517.82 1528.85 1550.06 1580.64 1585.92 1591.47 1597.39 1629.99 1638.47
1651.36 2232.66 3127.11 3180.69 3181.15 3183.33 3185.72 3189.46 3190.87 3194.32 3199.62
3203.25 3205.81 3210.66 3211.79 3214.92 3230.84

S6-13. The TS state for the TQB on the S_1 (Gaussian16)
Energy = -1428.452159 hartree

C	-0.07509400	-1.40239000	-0.00312800
C	-1.28537100	-0.65336100	0.01073300
C	-1.21239100	0.77582300	-0.00162200
C	0.03765900	1.44124000	0.01227400
C	1.21499800	0.65109500	-0.00043100
C	1.19206500	-0.76961100	0.01515500
N	-0.12845000	-2.76213600	-0.00496100
C	0.97809700	-3.57831200	-0.00239300
C	2.26865900	-2.99401100	-0.00056200
C	2.41270300	-1.55254300	0.00447900
C	3.38632500	-3.84264400	0.00104900
C	3.22928100	-5.22135300	-0.00113900
C	1.95007800	-5.78413600	-0.00398900
C	0.82516200	-4.96761600	-0.00525100
N	2.43884200	1.25161100	0.00075200
C	2.63239700	2.59052300	-0.00048700
C	1.50699900	3.45493600	-0.00266800
C	0.15564000	2.89630700	0.00087300
C	1.72357900	4.83355100	-0.00363900
C	3.01278300	5.34794600	-0.00434300
C	4.12071200	4.48521600	-0.00315100
C	3.94005400	3.11827500	-0.00168500
N	-2.36425200	1.50602000	0.00038900
C	-3.61660600	0.96410000	0.00049200
C	-3.76975400	-0.43876100	-0.00075800
C	-2.59544200	-1.30677000	0.00152200
C	-5.06239600	-0.98180100	-0.00126800
C	-6.17385900	-0.16183100	-0.00104300
C	-6.00937900	1.23269900	-0.00026800
C	-4.75017100	1.79601500	0.00038800
O	3.56679900	-1.00934600	0.00449600
O	-0.84503100	3.64631500	-0.00223400
O	-2.72127000	-2.54985700	-0.00200300
H	-1.07932400	-3.14619900	-0.00602200
H	4.36695400	-3.38841300	0.00371300
H	4.10050800	-5.86359700	0.00000400
H	1.82928900	-6.85949000	-0.00579300
H	-0.17027100	-5.39356000	-0.00765300
H	3.21738700	0.53086600	0.00234700
H	0.86230000	5.48719700	-0.00435800
H	3.16554100	6.41885700	-0.00558400
H	5.12247000	4.89355800	-0.00377300
H	4.78341500	2.44015400	-0.00079500
H	-2.21855500	2.51963400	0.00026300
H	-5.15883700	-2.05875400	-0.00166000

H	-7.16753300	-0.58918400	-0.00147600
H	-6.87927700	1.87704700	-0.00019200
H	-4.62139800	2.87100800	0.00080500

Corresponding Frequencies (in cm^{-1}):
-85.96 37.10 39.03 41.62 80.76 92.42 95.29 112.51 128.00 149.52 150.18 209.73 228.54 242.29 248.85 258.42 273.07 274.74 288.62 302.53 316.09 342.74 376.14 403.58 406.15 411.65 420.21 485.16 492.80 495.35 497.55 500.07 514.96 529.60 534.64 548.43 551.79 582.79 597.97 603.98 612.86 620.49 634.32 645.33 648.81 650.18 658.51 689.42 702.13 707.30 739.92 748.13 759.70 761.49 765.89 813.76 832.94 835.19 836.10 851.30 852.37 853.80 861.61 882.67 891.68 917.04 956.47 961.80 962.30 963.52 973.79 976.37 1001.82 1029.14 1031.44 1037.32 1053.90 1060.86 1087.28 1123.85 1132.59 1139.49 1163.04 1172.55 1184.25 1185.13 1192.89 1216.65 1223.69 1242.64 1258.10 1278.71 1285.35 1295.82 1299.81 1319.74 1339.80 1343.89 1353.67 1361.51 1371.20 1376.28 1397.93 1408.99 1426.30 1446.44 1475.91 1489.16 1495.97 1511.27 1514.04 1517.22 1526.32 1555.03 1564.92 1577.70 1585.34 1607.13 1610.85 1620.25 1629.60 1632.54 1640.17 2689.55 3179.24 3180.43 3186.90 3190.13 3191.45 3198.56 3200.59 3200.90 3207.94 3213.26 3214.98 3215.69 3281.84 3309.77

S6-14. The TQB-TB in S_1 state (Gaussian16)
Energy = -1428.467334 hartree

C	0.19513600	-1.37386200	-0.00405000
C	-1.15118200	-0.86501300	0.01512200
C	-1.34437100	0.52377200	-0.00148900
C	-0.21662700	1.42375800	0.01103800
C	1.08759000	0.89201100	0.00139400
C	1.31165200	-0.53224900	0.01905900
N	0.37859200	-2.72871000	-0.00878900
C	1.61298400	-3.34124300	-0.00438100
C	2.77634400	-2.52556800	0.00008300
C	2.62081400	-1.11743500	0.00555500
C	4.03781800	-3.15957300	0.00324500
C	4.13054000	-4.54450400	-0.00112300
C	2.97993800	-5.32994400	-0.00675800
C	1.71927600	-4.72839500	-0.00956800
N	2.19523700	1.68292800	0.00133200
C	2.09062800	3.01761700	-0.00122800
C	0.83331800	3.68172600	-0.00273200
C	-0.38705700	2.87832100	0.00068800
C	0.78730700	5.07150400	-0.00479100
C	1.96343000	5.81450700	-0.00673500
C	3.20971600	5.16969600	-0.00589000
C	3.28067800	3.79566600	-0.00350400
N	-2.59598600	1.04927800	0.00004000
C	-3.73266500	0.29126200	0.00085100
C	-3.62428400	-1.11185100	-0.00104100
C	-2.31478700	-1.75389500	0.00210500
C	-4.80071600	-1.88228400	-0.00157500
C	-6.03679700	-1.27732100	-0.00100200
C	-6.13070500	0.12699600	0.00006700
C	-4.99700500	0.90704300	0.00075600
O	3.75128500	-0.38345300	0.01301500
O	-1.50592600	3.42733100	-0.00268300

O	-2.21770400	-2.99851900	-0.00196500
H	-0.48722600	-3.27453200	-0.00936200
H	4.92505600	-2.54404600	0.00839300
H	5.10514000	-5.01578700	0.00103900
H	3.05615700	-6.40923600	-0.01023700
H	0.81893500	-5.32960700	-0.01391300
H	3.46531500	0.56828700	0.01146900
H	-0.17790800	5.55933700	-0.00536800
H	1.91646400	6.89522100	-0.00878000
H	4.11827400	5.75716800	-0.00743300
H	4.23030400	3.27787800	-0.00306100
H	-2.63797700	2.07015500	-0.00082300
H	-4.69913700	-2.95866000	-0.00204300
H	-6.93760000	-1.87657000	-0.00115000
H	-7.10394000	0.60086700	0.00033700
H	-5.06384600	1.98772300	0.00133700

Corresponding Frequencies (in cm^{-1}):
28.52 34.37 38.64 74.06 83.55 88.67 119.90 121.46 125.75 139.81 147.85 211.05 238.60 247.21 257.85 274.39 278.95 279.77 295.81 322.25 328.02 359.82 377.01 390.10 397.35 414.37 428.44 498.83 500.98 502.03 506.96 516.55 521.45 539.14 550.37 553.52 576.48 592.99 602.12 616.66 630.54 637.08 648.79 649.61 653.06 662.21 664.04 704.24 713.70 727.65 738.45 766.45 766.86 768.07 775.25 812.24 827.57 831.91 841.18 854.94 860.15 876.22 877.50 886.11 893.54 905.54 907.84 946.36 968.02 974.51 980.22 983.33 984.62 1024.01 1026.13 1050.87 1054.80 1062.64 1120.03 1128.22 1141.55 1161.40 1171.50 1179.92 1183.65 1186.15 1202.67 1228.37 1242.85 1263.10 1267.55 1282.52 1290.61 1302.56 1328.18 1342.13 1347.31 1358.10 1369.00 1384.02 1390.49 1394.66 1409.66 1441.29 1448.47 1473.16 1482.45 1485.11 1500.88 1510.39 1514.99 1528.47 1553.72 1564.12 1567.02 1586.60 1596.32 1599.61 1614.99 1627.04 1632.95 1641.08 1655.30 3177.40 3180.04 3184.46 3186.57 3190.32 3191.90 3200.31 3200.39 3201.79 3209.62 3211.76 3214.84 3221.11 3322.69 3357.63

S6-15. The TQB-TB in S_1 state (ORCA).
Energy = -1428.472395433113 hartree

C	0.19371464168247	-1.37554545722234	-0.00530158938485
C	-1.15175670737550	-0.86646564346220	0.01325809424801
C	-1.34546536381091	0.52443577368307	-0.00268324032149
C	-0.21936730054927	1.42372081372317	0.00960814848038
C	1.08528501714811	0.89158761358082	0.00082842329840
C	1.31052310699674	-0.53236889531525	0.01849216943270
N	0.37954786094576	-2.73024183096618	-0.01015521945132
C	1.61489802611110	-3.34096176310949	-0.00491886600037
C	2.77758420277675	-2.52411265433377	0.00050912239019
C	2.61991227548105	-1.11587548854667	0.00592293706419
C	4.03965887405554	-3.15767759452461	0.00448076041012
C	4.13369705794053	-4.54291607641486	-0.00010148013072
C	2.98325852820948	-5.32937511180221	-0.00667469678512
C	1.72217527461040	-4.72826011761198	-0.01021762292820
N	2.19254794883022	1.68197258927412	0.00138034014247
C	2.08925664322555	3.01675759162277	-0.00115977329788
C	0.83234928829939	3.68167684229565	-0.00319817840283
C	-0.38862854579238	2.87863632258264	-0.00054537040129
C	0.78848716163418	5.07166054576949	-0.00515631854439

C	1.96602213410561	5.81324999813357	-0.00656665861290
C	3.21164633765981	5.16708033577645	-0.00518824920736
C	3.28069325323292	3.79255989498943	-0.00281262285687
N	-2.59763109134776	1.04797200955996	-0.00087690519858
C	-3.73329284243462	0.29017389932820	0.00054876425478
C	-3.62566663582267	-1.11310941345618	-0.00124661296339
C	-2.31618251476445	-1.75442271563771	0.00086810126118
C	-4.80372618309371	-1.88215773473667	-0.00084998630616
C	-6.03931928303253	-1.27564677907378	0.00058126335818
C	-6.13183304249135	0.12935853608407	0.00151415871861
C	-4.99700693906995	0.90792874842513	0.00124009349927
O	3.75047667750326	-0.38000692833379	0.01386322054014
O	-1.50754439964575	3.42801550475377	-0.00363173097778
O	-2.21894997729770	-2.99998277510019	-0.00351171124454
H	-0.48290615848627	-3.28040006662093	-0.01116721751740
H	4.92757142305058	-2.54307261355134	0.01035338219369
H	5.10876361737811	-5.01321201034441	0.00265396433898
H	3.05997540940129	-6.40867100439501	-0.01029126974432
H	0.82141365618088	-5.32893532175051	-0.01524472517812
H	3.46478620074310	0.57253064210104	0.01122445496065
H	-0.17508104073762	5.56285872469047	-0.00609568879208
H	1.92021729508717	6.89407900524686	-0.00856733095598
H	4.12101541339071	5.75346449140016	-0.00627501675168
H	4.22938719047939	3.27292636259144	-0.00182337062777
H	-2.64497187910313	2.06823021351666	-0.00179967753145
H	-4.70596802183669	-2.95887498280513	-0.00125012661348
H	-6.94073420805739	-1.87403724360214	0.00122184640340
H	-7.10464456230956	0.60423265084153	0.00246117290270
H	-5.05970581910091	1.98901611274691	0.00175183883027

Corresponding Frequencies (in cm^{-1}):
24.69 31.10 35.59 70.32 81.90 89.67 119.28 121.21 126.03 138.93 146.28 211.37 238.22 248.07 258.08 274.89 278.87 279.64 295.19 323.12 328.34 358.90 377.73 391.31 396.81 413.25 431.06 498.43 499.67 501.56 506.90 515.39 519.91 539.90 549.30 553.43 575.54 592.90 600.59 615.82 629.76 635.92 648.42 649.00 653.24 661.50 661.74 703.88 713.91 727.62 736.51 764.26 765.13 765.51 774.04 794.46 815.97 826.48 840.38 844.55 861.47 870.84 876.74 888.99 893.98 898.70 907.15 946.84 967.37 974.07 980.53 984.21 987.74 1022.44 1024.84 1050.70 1053.85 1060.87 1118.38 1127.37 1141.27 1159.33 1170.14 1178.21 1180.76 1184.60 1201.24 1227.50 1242.04 1260.48 1264.66 1279.50 1291.26 1300.95 1327.34 1339.87 1345.23 1357.28 1368.16 1381.24 1389.70 1394.46 1406.90 1436.79 1445.47 1471.39 1480.13 1483.22 1497.77 1506.50 1512.54 1524.66 1550.40 1562.00 1564.32 1581.78 1593.13 1597.03 1611.28 1623.52 1629.87 1635.97 1651.31 3171.16 3177.20 3179.56 3184.18 3189.21 3189.93 3198.57 3198.95 3199.82 3207.93 3211.55 3212.04 3218.35 3327.38 3366.37

S6-16. The TQB-TB in T_1 state (ORCA).
Energy = -1428.485363415613 hartree

C	0.19030347531314	-1.37544796331479	0.00443045048559
C	-1.14513504612256	-0.86137423710570	0.01864341469961
C	-1.33360386922430	0.54177828552114	0.00927924416610
C	-0.22420749912996	1.43371104609212	0.02230396939238
C	1.08642340928888	0.88686193377904	0.00955666090256
C	1.31595575917147	-0.51189687263933	0.03055163344248

N	0.37469665127954	-2.71031042782844	-0.00282544041578
C	1.61077866857678	-3.33530692905064	-0.00246024420064
C	2.77312055022238	-2.52141246261748	0.00304536388007
C	2.62686462794037	-1.12181916141872	0.01419510396728
C	4.03325767768047	-3.17025018194899	0.00174696067422
C	4.11278141480349	-4.55003521865721	-0.00882810275781
C	2.95459422560128	-5.33267161869038	-0.01616721799250
C	1.70183733068790	-4.71916300200859	-0.01419863600665
N	2.21208377433648	1.69377817788917	0.01177319471985
C	2.09953143228129	3.01926626362663	0.00413316653100
C	0.83339414776153	3.68321325979913	-0.00418409501410
C	-0.39843255685337	2.87124147122627	0.00416898256362
C	0.78571294088945	5.06569803809110	-0.01400814617647
C	1.96670148977988	5.81890705648613	-0.01888037767646
C	3.21606567338177	5.18042046975826	-0.01174174093386
C	3.29243851942727	3.80597067753382	-0.00069804168305
N	-2.59197455392409	1.05002732809562	0.00805846580388
C	-3.72766483784234	0.28284871951207	0.00386429259571
C	-3.61949736636037	-1.11748300299372	0.00127590733020
C	-2.30156544707490	-1.75131369253319	0.00428955881951
C	-4.78940312990860	-1.89273490183465	-0.00401475881378
C	-6.03052309608690	-1.29198940621535	-0.00620281819125
C	-6.12599444690300	0.10882560467198	-0.00362884657622
C	-4.99222564069780	0.89366527185309	0.00136391300944
O	3.75392246754994	-0.38147010280807	0.02440289016250
O	-1.51225971261218	3.43506620143043	-0.00083037199088
O	-2.19983644515176	-2.99595029000980	-0.00263446897293
H	-0.49258698574676	-3.25847521322762	-0.00708912096181
H	4.92666499825027	-2.56459421334615	0.00775767954719
H	5.08363250419985	-5.02824354648459	-0.01030552227986
H	3.02353508199806	-6.41134668352189	-0.02470411304446
H	0.79539573435652	-5.31064365059752	-0.01992374669672
H	3.46831916672547	0.57130632287322	0.02276597977420
H	-0.18006312315194	5.55371706977756	-0.01870315730607
H	1.91348605265181	6.90000132720738	-0.02763785572972
H	4.12243029070931	5.77241934795925	-0.01561555359643
H	4.24356433727399	3.28929578509681	0.00457643402641
H	-2.64290969677121	2.07186563046708	0.00691942862670
H	-4.68594631795874	-2.96902800653579	-0.00589300453561
H	-6.92887675896567	-1.89474869230325	-0.01001041875492
H	-7.09995906279349	0.58144031791485	-0.00549977207722
H	-5.06034480885861	1.97417887102968	0.00303387726470

Corresponding Frequencies (in cm^{-1}):
31.45 33.97 39.34 73.57 91.37 93.99 120.08 120.77 127.60 142.71 150.03 213.50 240.57 256.99 257.66 275.64 277.22 281.00 297.57 320.90 330.00 356.14 375.04 398.61 402.44 410.99 427.00 485.94 489.16 503.31 505.68 512.44 521.97 532.43 547.21 551.73 589.07 590.99 610.32 615.94 628.72 631.63 639.62 645.73 649.44 651.86 662.62 702.95 715.65 731.12 739.30 757.61 760.97 770.21 771.09 817.11 828.41 831.71 837.41 847.52 861.50 865.31 880.88 896.03 901.98 905.97 925.79 958.97 969.27 972.25 972.31 975.51 984.75 1019.34 1022.03 1048.55 1056.55 1062.39 1114.99 1125.78 1138.92 1145.21 1163.69 1169.27 1178.04 1181.84 1184.89 1234.71 1238.24 1262.19 1270.17 1281.02 1291.62 1306.72 1328.62 1333.23 1336.60 1351.26 1356.97

1376.18 1386.49 1394.92 1409.59 1424.79 1446.05 1465.79 1474.93 1478.92 1490.83 1513.80
1522.58 1535.30 1541.88 1560.36 1567.44 1584.45 1586.66 1598.56 1603.36 1615.37 1634.57
1643.58 1655.77 3144.95 3181.10 3183.38 3183.60 3188.59 3190.29 3193.42 3199.50 3201.31
3205.57 3207.57 3210.13 3223.83 3270.40 3333.58

S6-17. The TQB-TB in T_2 state (ORCA)
Energy = -1428.471999374666 hartree

C	0.17232865624201	-1.40608931637829	0.00953797976551
C	-1.14481952015034	-0.89216524016354	0.02600470486254
C	-1.32469097138584	0.51604520640366	0.01099767966663
C	-0.21560279768918	1.42390054675893	0.02732525984520
C	1.09015227963225	0.88492583650593	0.00931744253248
C	1.30379627922796	-0.53957126248508	0.02146020453743
N	0.37423638794215	-2.75757648847113	0.01084618569486
C	1.59775024132082	-3.33903294305947	0.00404704061158
C	2.75916274889665	-2.52452080280809	-0.00261972701846
C	2.59428355187732	-1.10342220469182	0.00271761808198
C	4.02564914775784	-3.13739116921038	-0.01118507861339
C	4.13705694531706	-4.51232229978084	-0.01392908896223
C	2.97882360000020	-5.31202015578210	-0.00777551757165
C	1.72544550266656	-4.74334164854966	0.00087351697221
N	2.19373056422902	1.66764831429921	0.00604793273294
C	2.09153186917817	3.01949656143243	0.00373783195527
C	0.82577902224411	3.67074568190386	0.00616820949372
C	-0.38225715089463	2.86908531142898	0.01744483655258
C	0.79168340532221	5.06693970480599	-0.00051757386206
C	1.98375961013661	5.81066609973753	-0.01108087584047
C	3.21958741081445	5.17580707797168	-0.01309548902966
C	3.27925358410449	3.78184441496778	-0.00622286565163
N	-2.57552328120928	1.03856824472589	0.00784269697044
C	-3.71149382704026	0.28605420314500	0.00128946176986
C	-3.61581176717485	-1.12362695646163	0.00150494663917
C	-2.31558427192764	-1.76965040900058	0.01407751647374
C	-4.80105183205297	-1.88311202672787	-0.00767426582328
C	-6.03167951838587	-1.26723282330773	-0.01728965655159
C	-6.11321641310732	0.13962670085000	-0.01752239486644
C	-4.97309629620132	0.91006433013234	-0.00850774072765
O	3.68302802930352	-0.35983410246849	-0.00381715230053
O	-1.53248845684255	3.41806654137009	0.01930116506862
O	-2.20831756565065	-3.01927589478256	0.01493440123958
H	-0.50162440270953	-3.30332312090368	0.01325465934591
H	4.90495937107906	-2.50940366638292	-0.01565178536394
H	5.11307209497786	-4.97809826583728	-0.02067855938926
H	3.07282518879031	-6.39031520529911	-0.01018069772370
H	0.83187678025826	-5.35399253933568	0.00533836515644
H	3.36137299135242	0.61857648253251	0.00003566942077
H	-0.16876147225963	5.56258925781553	0.00132442653192
H	1.93340569241920	6.89249204441663	-0.01733113432444
H	4.13289795427259	5.75554708411973	-0.02113184570683
H	4.22699751046469	3.25919984618466	-0.00842643790061
H	-2.59329582517027	2.07177907038699	0.01067644961263

H -4.71331851748746 -2.96075387393472 -0.00691071428489
H -6.93763553320240 -1.85858697997093 -0.02450518830457
H -7.08250248794874 0.62140312454405 -0.02528276347011
H -5.02719251133699 1.99138270935413 -0.00931864824662
Corresponding Frequencies (in cm^{-1}):
30.82 35.40 41.56 80.14 92.91 99.80 118.37 120.01 130.49 147.48 152.47 214.36 246.21
254.81 255.70 272.15 278.15 284.47 302.16 317.88 332.24 360.24 373.48 401.04 403.02 424.69
427.29 498.39 500.38 502.96 505.56 506.80 519.75 546.98 557.03 560.32 578.94 590.32 607.74
617.59 624.29 631.25 644.90 649.88 650.74 657.74 668.45 682.13 703.25 727.44 739.61 756.03
761.78 767.28 768.47 825.80 835.20 846.13 857.14 875.09 883.30 885.84 897.11 899.01 900.56
903.10 948.48 959.97 967.54 969.35 975.11 982.01 1010.11 1014.30 1031.04 1036.31 1048.52
1063.30 1101.12 1130.41 1138.20 1141.17 1169.84 1175.03 1179.38 1183.62 1193.02 1205.41
1220.67 1248.60 1266.04 1284.42 1296.27 1303.52 1316.42 1346.98 1351.57 1362.81 1374.16
1376.18 1381.79 1387.17 1393.85 1438.48 1449.18 1453.11 1469.98 1481.03 1492.22 1503.38
1513.86 1521.96 1525.60 1542.49 1561.79 1562.27 1572.27 1610.31 1620.12 1622.38 1628.46
1637.56 2029.80 2587.22 3173.73 3178.93 3180.94 3185.42 3189.80 3190.64 3195.87 3199.23
3201.58 3203.25 3203.68 3210.83 3211.60 3217.42

S6-18. The TQB-TB in T_3 state (ORCA).
Energy = -1428.465532987547 hartree
C 0.18513978685875 -1.38491320929653 0.00703852700909
C -1.14754836974735 -0.87969460525786 0.02223647695466
C -1.32319320852850 0.51885809912951 0.00954894779045
C -0.20916231167893 1.42600255117286 0.02824469369797
C 1.09676200041267 0.89047277690628 0.00961776125213
C 1.30185309696084 -0.53094181189187 0.02485970630833
N 0.38254178249885 -2.73782867726367 0.00831963423688
C 1.60475408367660 -3.33212262567859 0.00407560268738
C 2.76926142202567 -2.52212261203035 -0.00057575913207
C 2.61454809411981 -1.11060677236410 0.00626819513661
C 4.02908782448592 -3.15690151943292 -0.00897410119405
C 4.12053162645912 -4.55223031032819 -0.01317707785780
C 2.97112631409918 -5.33490440827527 -0.00768762884491
C 1.71015865469740 -4.73397013327938 0.00014595078773
N 2.20868579335544 1.66544365487195 0.00747671197238
C 2.09552575313502 3.01372375568878 0.00425112765293
C 0.82794140112800 3.66380010614583 0.00574122873943
C -0.38579959937287 2.86760736375685 0.01691754957109
C 0.78138055898900 5.06332855788502 -0.00298654785381
C 1.95738941499396 5.81489453542678 -0.01339851543888
C 3.20109677087489 5.18030896939429 -0.01377324069660
C 3.27637221065197 3.79500721398917 -0.00525057719000
N -2.56947138777641 1.04375181879126 0.00640613941087
C -3.70717718758301 0.28661374845564 0.00037691580726
C -3.60879947382974 -1.11994845040956 -0.00135678154990
C -2.30025135292413 -1.76191316763413 0.00706743959303
C -4.78874354838920 -1.88295645430144 -0.00856491945600
C -6.02206981237688 -1.26940150554318 -0.01436475807311
C -6.10709261042055 0.13474109419188 -0.01292554655689
C -4.96818389652711 0.90861601063676 -0.00593977479582
O 3.69501257939091 -0.35239471170776 0.00303369942688

O	-1.53039477760950	3.41415975154447	0.01815881650729
O	-2.19177256602756	-3.01272412156890	0.00491793552466
H	-0.50240742409767	-3.27682568234092	0.00752258841330
H	4.91983496588139	-2.54581658083913	-0.01261141229279
H	5.09511612098327	-5.02204544012558	-0.02010355261617
H	3.04598830080534	-6.41377709318160	-0.01060316331372
H	0.80726631730169	-5.33075937327646	0.00346543752357
H	3.35183348154058	0.63152425605417	0.00513542855173
H	-0.18593725258003	5.54591251826634	-0.00214041812382
H	1.90290300448558	6.89615617651603	-0.02083887383938
H	4.10979872132359	5.76825292235742	-0.02170020495170
H	4.23026385099959	3.28378676257125	-0.00644693613399
H	-2.58356302926251	2.07926540408804	0.00980439787129
H	-4.69755956765763	-2.96034841806048	-0.00922585198493
H	-6.92640420146468	-1.86337724348996	-0.01977049602571
H	-7.07754391241642	0.61431038380714	-0.01758352089623
H	-5.02461644186427	1.98978149593006	-0.00518025360870

Corresponding Frequencies (in cm^{-1}):

29.94 36.69 39.57 80.79 96.19 97.17 116.14 125.75 133.26 149.45 151.77 212.18 246.07 254.08 255.60 274.03 283.91 285.97 306.55 325.96 328.87 358.00 377.80 398.01 402.10 413.06 430.52 498.78 502.38 504.21 509.99 510.22 521.84 554.61 559.94 575.99 581.17 592.98 612.16 617.48 630.97 635.81 646.08 651.65 652.88 658.71 668.87 703.52 705.95 727.94 733.20 741.85 766.32 767.72 777.03 806.34 829.89 839.29 859.74 879.56 896.10 896.70 897.51 902.58 907.14 919.33 943.57 947.86 948.40 969.63 970.21 985.30 1010.81 1025.69 1037.23 1050.63 1058.18 1069.23 1108.38 1115.12 1140.00 1159.42 1165.09 1178.03 1182.00 1194.16 1203.71 1225.80 1235.31 1244.47 1269.86 1281.09 1293.15 1309.85 1326.43 1333.38 1359.45 1368.29 1373.01 1379.58 1385.99 1406.14 1416.43 1437.70 1454.98 1460.20 1478.30 1483.74 1496.15 1502.57 1514.67 1520.83 1530.67 1538.66 1544.70 1575.31 1580.44 1591.48 1615.97 1628.05 1631.15 1650.53 2285.96 2391.01 3126.96 3141.26 3178.72 3181.32 3188.77 3189.94 3191.02 3196.07 3199.62 3200.17 3208.26 3210.84 3210.97 3222.76
